# Supplementary material for: Pathophysiological Mechanisms in Long COVID: A Mixed Method Systematic Review
Source: Int J Environ Res Public Health. 2024 Apr 12;21(4):473. doi: 10.3390/ijerph21040473 (PMC11050596; doi:10.3390/ijerph21040473)
Supplement: Supplementary file 1 [file ijerph-21-00473-s001.zip › Table S1_ Summary of findings in Review 1 . Draft 2. NDB edits 10.4.24.pdf]

**Table S1.** Summary of findings in Review 1.

| Systematic Reviews – Review 1, Part 1 | | | | | | | | | | | | | |
|---|---|---|---|---|---|---|---|---|---|---|---|---|---|
| **Study ID** | **Title** | **Country** | **Search Criteria** | **Aim of Study** | **Study Design** | **Start Date** | **End Date** | **Study Funding Sources** | **Population Description** | **Inclusion Criteria** | **Exclusion Criteria** | **Total No.of Studies Included** | **Notes** |
| **Salamanna 2021** [23] | *Post-COVID-19 Syndrome: The Persistent Symptoms at the Post-viral Stage of the Disease. A Systematic Review of the Current Data* | Italy | Databases: PubMed; Web of Science; Embase; Google Scholar.<br>PICO model used to formulate questions for this study:<br>1) studies that considered patients with long-term COVID-19 symptoms (Population)<br>2) studies where the primary aim was to evaluate long-term COVID-19 symptoms in mild, moderate, severe, and critical patients that have a follow-up of at least 14 days (Interventions)<br>3) studies with or without a control group (Comparisons)<br>4) studies that reported the long-term COVID-19 symptoms (Outcomes). | To assess the current evidence on the long-term symptoms in COVID-19 patients. | All study types reporting at least one long-term COVID-19 symptom. | Database inception | 15th February 2021 | IRCCS Istituto Ortopedico Rizzoli (Ricerca Corrente) | Mild, moderate, severe, and critical COVID-19 patients with at least one long-term COVID-19 symptom after at least 14 days of follow-up. | Any study that reported at least one long-term COVID-19 symptom after at least 14 days of follow-up. | - Unpublished reports<br>- Unspecified date/location of the study<br>- Suspicion of duplicate reporting<br>- Coronavirus strains other than COVID-19<br>- Ureported long-term COVID-19 symptoms<br>- Studies that only hypothesize post-COVID-19 sequelae. | 145 | Presents symptoms and mechanisms together in Table 1 under each study, so these needed to be distinguished in reporting the findings.<br>All study designs similarly presented together in Table 1; these needed to be distinguished. Studies also varied by duration since COVID-19 onset, hence difficult to compare prevalence and longevity of symptoms and mechanisms. |
| **Garg 2021[28]** | *The Conundrum of Long-COVID-19: A Narrative Review* | India | Search terms included: Long COVID-19, post-COVID, chronic COVID, post-COVID syndrome long-haul COVID, viral illness following COVID19 post-COVID illness, COVID recovery, predictors of Long COVID.<br>Databases searched: Google Scholar, PubMed, Web of science, bioRxiv, medRxiv, and ResearchGate, references from relevant articles, and | To review the epidemiology, etiopathogenesis, clinical manifestations, predictors, and management strategies in COVID-19 survivors in their convalescent/ recovery phase. | Cohort studies and cross sectional clinical reviews or questionnaires. | Not clear | 25th April 2021 | Not declared | Mixed | Not reported | Not reported | 212 | This review was a mix of hospitalised and non hospitalised patients with mild, moderate, severe covid. |

| | | | | | | | | | | | | | |
|---|---|---|---|---|---|---|---|---|---|---|---|---|---|
| | | | internet sources (WHO reports). | | | | | | | | | | |
| **Joshee 2022[30]** | *Long-Term Effects of COVID-19* | United States | PubMed search was completed using the terms: long-COVID, post-acute COVID-19, post-COVID condition, post-COVID sequelae, long-term sequelae of COVID-19. | To report up-to-date epidemiology, pathophysiology, clinical predictors, management recommendations, and unanswered questions/future directions for the systematic effects of long-term COVID-19 infections. | Mix of various study types including cohort studies (including retrospective), meta-analysis, prospective studies, observational studies, systematic reviews and case reports. | 2020 | 17th July 2021 | None declared | Total number of participants unclear. 253,869 from numbers provided - 5 studies did not specify number of participants. | Peer-reviewed studies in English published by July 17, 2021.<br>All studies are from adult populations unless specified. | Not specified. | Unclear. | None. |
| **Tesarz 2022[24]** | *Pain, the brain, and SARS-CoV-2: evidence for pain-specific alterations in brain-related structure - function properties* | Germany | Databases: Medline; Web of Science. | Provide an overview of the central nervous alterations in the brain described in the context of SARS-CoV-2 infection, focusing on findings with brain imaging. | No study design criterion. | 10/2019 | Not clear | Supported by: German Research Foundation (DFG) within the Collaborative Research Center and by the German Federal Ministry of Education and Research. | Not clearly specified. | Included all types of studies that investigated SARS-CoV-2-associated central nervous system changes in humans using brain imaging techniques. | Studies that were limited to examining changes in the peripheral nervous system and those that did not focus on human individuals (e.g., animal studies, cell cultures, etc.) were excluded. | 69 | None. |
| **Houben 2022[29]** | *The Impact of COVID-19 Infection on Cognitive Function and the Implication for Rehabilitation: A Systematic Review and Meta-Analysis* | Belgium | Search terms included a combination of the following: COVID-19: (COVID* OR SARS-CoV-2); COGNITION: (“cognit*” OR “memory” OR “attent*” OR “intellect” OR “executive funct*” OR “recognit*” OR “IQ” OR “problemsolving” OR “psychomotor speed” OR “mental flexib*” OR “choice react*” OR “emotionalbias” OR “planning” OR “response inhibition”). References from selected papers and from other relevant articles were screened for potential additional studies in accordance with the snowball principle. Search limited to journal articles in English. | To summarise the current level of evidence supporting the negative impact of COVID-19 infection on cognitive functions. Also to present and discuss the different potential interventions available in rehabilitation to try to decrease the risk of cognitive disorders after COVID-19 infection and restore optimal cognitive functions in patients presenting long COVID symptoms. | Cohort study | Not stated | April 2022 | No external funding | 94,103 participants included: 90,317 COVID-19 patients and 3786 control. Of 27 studies included, five (959 participants, 513 patients) included in a meta-analysis to quantify the impact of COVID-19 on cognitive (sub)functions. | Healthy adults with COVID-19 diagnosed using PCR.<br>Any outcomes related to cognitive disorders, loss of cognitive functions, and/or cognitive fatigue. | Studies with patients suffering from neuropsychiatric disorders before infection were not included. | 27 | The authors were unsure of follow-up for patients in 14 of the 27 studies so it is unclear whether those studies relate to Long Covid.<br><br>Most (n =20) ranked good quality according to the AHQR standards using the NOS. 7 remaining studies ranked as fair quality. |

| | | | | | | | | | | | | | |
|---|---|---|---|---|---|---|---|---|---|---|---|---|---|
| **Pierce 2022[32]** | *Post-COVID-19 Syndrome* | United States | PubMed; CINAHL; Web of Science; official scientific and public health websites (e.g., CDC, NIH). Used combinations of the following key words: post-COVID-19 syndrome, post-SARS-CoV-2, long COVID-19, long COVID-19 syndrome, and pathophysiology of post-COVID-19. | To summarise and evaluate post-COVID-19 syndrome from a biological perspective: describe the symptoms and various definitions and categories of post-COVID-19 syndrome; discuss the known pathophysiological changes that occur in post-COVID-19 syndrome and possible mechanisms for these symptoms. | No criterion on study design. | Not stated | 30th August 2021 | Not reported | Not reported | Related to the pathophysiology of post-COVID-19 syndrome. | Articles on children aged <18 years and articles on animals. | 54 | Data were analysed using the constant comparison method. Design of included studies not reported: "many of the articles used retrospective and case control designs". |
| **Piri 2021[35]** | *A systematic review on the recurrence of SARS-CoV-2 virus: frequency, risk factors, and possible explanations* | Iran | Databases: Pubmed; Medline. | To evaluate rate of SARS-CoV-2 recurrence, changes in disease severity, the interval between negative RT-PCR test for SARS-CoV-2 and disease recurrence, disease and patients characteristics, risk factors for predicting recurrence and outcomes, as well as the prognosis of recurrent patients. | Any study reporting primary data. | Not reported | 20th November 2020 | None reported | 1,128 patients with at least one-time recurrence of SARS-CoV-2. | Only journal articles published in English, those with the original data, and only human studies related to recurrence of SARS-CoV-2 in previously recovered patients were included. | No exclusion criteria. | 66 | 18 cohort studies.<br><br>On re-infection but related regarding viral persistence. |
| **Willi 2021[25]** | *COVID-19 sequelae in adults aged less than 50 years: A systematic review* | Switzerland | Databases: Embase; WHO; Scopus; PubMed; Litcovid; bioRxiv; medRxiv. Search terms: "COVID-19", "coronavirus disease 2019", "SARS-CoV-2", "sequelae" and "consequence*"; "child*" and "pregnant"served as negative qualifier. | To evaluate the available evidence of all intermediate and long-term COVID-19 sequelae affecting formerly healthy adults aged 18-50 years. | No restriction Study types included prospective (n=11) and retrospective (n=11) cohort studies, cross-sectional studies (n=4), and case reports (n=5). | Not specified | 15th September 2020 | Maxi Foundation (Zurich) and MilMedBiol. | Adults aged 18-50 years with confirmed SARS-CoV-2 infection. Both sexes and formerly healthy participants as well as participants at risk were included. | - Studies including original data and systematic and narrative reviews<br>- English language publications, including preprints<br>- Subjects with confirmed SARS-CoV-2 infection<br>- Both sexes and formerly healthy participants as well as participants at risk. | - Articles including children (<18 years) or only elderly (>50 years)<br>- Animal, laboratory or in vitro studies. | 31 | Risk of bias assessment performed using a modified Newcastle-Ottawa Scale for assessing the quality of non-randomised studies (except case reports): "very good" for n=4; "good" for n=5; "satisfactory" for n=12; "unsatisfactory" for n=5; no rating for case reports n=5. |
| **Ramadan 2021[33]** | *Cardiac sequelae after coronavirus disease 2019 recovery: a systematic review* | Italy | Retrospective and prospective studies, case series, cross-sectional studies and case reports that described adult patients who underwent any type of | To assess the range of cardiac sequelae after COVID-19 recovery. | Prospective cohort (15), case reports (7), cross-sectional design (5), case series (4), retrospective cohort (3), ambidirectional cohort (1). | 2019 | 2021 | None. | Total: 52,609 Breakdown unclear but mostly hospitalised: Twenty-two articles (63%) included patients previously hospitalized for | - Adult patients (18 years+) post COVID-19 recovery<br>- SARS-CoV-2 infection, diagnosed by PCR<br>- Cardiac sequelae after recovery including symptoms, functional or | - Under 18 years old<br>- Cardiac manifestations before recovery (during acute infection. | 35 | From paper: "Included studies were observational in nature, and most had a fair risk of bias, the latter being primarily due to absence of a comparison group in cohort studies". |

| | | | | | | | | | | | | | |
|---|---|---|---|---|---|---|---|---|---|---|---|---|---|
| | | | cardiac assessment after COVID-19 recovery.<br><br>Databases: PubMed, Embase, Scopus and Google scholar. | | | | | | COVID-19 (n = 51,117, 97.2%)<br>Eight studies contained a mixed population of discharged patients and outpatients (n =1330, 2.5%)<br>Five included only outpatients (n = 122; 0.3%)<br>(NOTE: The patient numbers do not add up to 52609 although the percentages add up to 100%.) | structural changes. | | | |
| **Michelen 2021[7]** | *Characterising long COVID: a living systematic review* | UK | Living systematic review updated around every 6 months.<br>Databases: Medline; CINAHL; Global Health (Ovid); WHO Global Research Database on COVID-19; LitCovid; Google Scholar (first 500 hits on 17 Mar 2021); references of systematic reviews.<br>(Risk of bias assessment: 12 studies were at high risk of bias, 22 at moderate risk, 5 at low risk.) | To regularly synthesise evidence on long COVID characteristics, to help inform clinical management, rehabilitation strategies and interventional studies to improve long-term outcomes. | Any study with 100+ COVID patients followed up for 12+ weeks post-onset. Included cohort studies (82%), cross-sectional studies (15%), and case-control study (3%). | 1st January 2020 | 17th March 2021 | UK Foreign, Commonwealth and Development Office and Wellcome , the Bill & Melinda Gates Foundation and the EU FP7 project PREPARE. | 10,951 (range: 100-1,733) patients in 12 countries, aged from 9 months to 93 years old; 48% female. 78% of patients were hospitalised during acute phase.<br>67% (26/39) of studies were cohorts of hospitalised patients post discharge.<br>10% (4/39) followed up people who were not hospitalised.<br>23% (9/39) included both.<br>56% (22/39) included ICU patients. | - Peer-reviewed studies<br>- Involved at least 100 people with laboratory confirmed and/or clinically diagnosed COVID-19<br>- Studies that reported symptoms or outcomes at 12+ weeks post COVID-19 onset<br>- No language restrictions. | - Reviews and opinion pieces<br>- Studies with <100 participants<br>- Follow-up unclear or <12 post-onset<br>- Non-peer-reviewed preprints. | 39 | No case reports or case series. Only 4 studies included a control group. |
| **Renaud-Charest 2021[26]** | *Onset and frequency of depression in post-COVID-19 syndrome: A systematic review* | Canada | Databases: PubMed; Medline; Google scholar.<br>Search string used for PubMed and OvidMedline: ("depression" OR mood disorders) AND ("COVID-19" OR "SARS-CoV-2" OR "Coronavirus disease 2019") AND (follow up OR prospective) AND (sequelae OR recover* OR surviv*).<br>A more restrictive search string was used for Google Scholar: "depression" AND | To determine the frequency of depressive symptoms and clinically significant depression more than 12 weeks following SARS-CoV-2 infection. | Case-control studies, cohort studies, uncontrolled observational studies, cross-sectional studies, and retrospective chart reviews. | 1st January 2020 | 5th June 2021 | Unclear. | Mix of hospitalised (ICU or no ICU admission) and non-hospitalised patients. | - English language articles<br>- Case-control studies, cohort studies, uncontrolled observational studies, cross-sectional studies, and retrospective chart reviews<br>- SARS-CoV-2 infection confirmed by reverse transcriptase polymerase chain reaction (RT-PCR) of nasopharyngeal or saliva swabs, or confirmed by antibody assays of blood<br>- Depressive symptoms and clinically-significant | - Animal studies<br>- Case reports, unpublished data sets, review articles, metaanalyses<br>- SARS-CoV-2 infection not confirmed by RT-PCR of nasopharyngeal or saliva swabs, or antibody assays of blood<br>- Depressive symptoms and clinically-significant depression in the general population reported as socioeconomic consequences of the COVID-19 pandemic. | 8 | Six uncontrolled observational studies and two prospective cohort studies.<br><br>Methodological quality and risk of bias assessed by Newcastle-Ottawa Scale: five studies had high NOS scores; three had moderate scores. |

| | | | | | | | | | | | | |
|---|---|---|---|---|---|---|---|---|---|---|---|---|
| | | | "psychiatric" AND "mental health" AND "after COVID-19" AND "virus" AND "follow up" AND "sequelae". Additionally, reference lists of included articles and identified reviews on the COVID-19 neuropsychiatric sequelae were manually reviewed. | | | | | | | depression assessed using a validated and standardized scale (e.g., HADS-D, PHQ-9) or criteria from the Diagnostic and Statistical Manual of Mental Disorders (DSM-IV or DSM-V), and depressive symptoms and clinically-significant depression assessed more than 12 weeks following SARS-CoV-2 infection. | | | |
| **Hussain 2022[20]** | *A systematic review of acute telogen effluvium, a harrowing post – COVID-19 manifestation* | The Netherland | MEDLINE/PubMed and Embase databases for studies published Dec 2019 - 5 Oct 2021. Search terms included: "Coronavirus", "Covid-19", "SARS-CoV-2", "Telogen effluvium", "TE", "Hair loss", "Hair fall", "Scalp" and "latemanifestation" | To compile and illustrate the clinical characteristics, physical examination findings, outcomes, and possible pathology behind acute Telogen Effluvium occurring in COVID-19 recovered patients. | Case series | December 2019 | 5th October 2021 | None. | 632 patients (39% hospitalised) with a history of COVID-19 infection were included from 19 studies.<br>Of these 465 patients reported hair loss after recovery from COVID-19 and were subsequently diagnosed with acute telogen effluvium. | - Adult participants 18 years of age<br>- Observational studies, case reports, and case series<br>- Studies reporting patients recovered from laboratory confirmed SARS-CoV-2 infection presenting with hair loss weeks to months after infection. | Review articles, posters, and duplicate publications. | 19 | 1. Systemic review conducted under PRISMA guidelines.<br>2. Quality assessment results of the 19 studies reported as:<br>'Fair' = 11<br>'Good' = 2<br>'7 (NCOS)' = 3<br>'8 (NCOS)' = 2<br>None given = 1<br>3. Limitations given:<br>- "these reports are underpowered as many cases of acute TE go unreported, and hence, the findings may not be decisive and relevant to the entire population";<br>- "most of the studies were case series that described patients selected for inclusion because they had acuteTE and therefore the incidence and prevalence of this complication post-COVID-19 cannot be determined";<br>- "although COVID-19 has been established as the main trigger for acute TE, other factors, including poor nutrition, deteriorating scalp health, infrequent shampooing, and medications such as anticoagulants, may be potential contributors. Moreover, the emotional toll of the COVID-19 pandemic on the mental health, of even the non infected individuals, may be an inciting factor for acute TE". |

| | | | | | | | | | | | | | |
|---|---|---|---|---|---|---|---|---|---|---|---|---|---|
| **Ceban 2022[36]** | *Fatigue and cognitive impairment in Post-COVID-19 Syndrome: A systematic review and meta-analysis* | Canada | Search string: "long covid" OR "persistent covid" OR "post covid" OR "post-acute sequelae of SARS-CoV-2 PASC" OR "enduring COVID-19 sequelae" OR "long-haul covid" OR "long-tail covid". | 1. Quantify proportion of individuals exhibiting fatigue and cognitive impairment 12+ weeks following COVID-19 diagnosis.<br>2. Characterize the inflammatory correlates and functional consequences of PCS. | 1. No restrictions on study design/type of article.<br>2. No language restrictions.<br>3. No publication date restrictions. | Not stated | 8th June 2021 | None | Hospitalised and Community cases. | - Confirmed Covid-19<br>- One or more ongoing symptoms 12+ weeks following diagnosis<br>- Primary research<br>- Full text articles (including preprints) | - Incomplete/inexact quantitative data<br>- Symptoms existed prior to acute COVID19 infection and did not markedly worsen at 12+ weeks follow-up after COVID19<br>- New symptoms following recovery from acute COVID19 infection<br>- Unconfirmed COVID19 infection<br>- Median/mean follow-up time of less than 12 weeks since acute COVID19<br>- Small studies ( n<10), protocols, case reports, post-mortem studies, abstracts, unpublished (preprints included if full text articles). | 81 | With the exception of PHSOP-COVID (~1000 patients), most of the studies were quite small (~100 patients). |
| **Sansone 2022[27]** | *The Sexual Long COVID (SLC): Erectile Dysfunction as a Biomarker of Systemic Complications for COVID-19 Long Haulers* | Italy | Databases: PubMed, Scopus and Google Scholar through July 2021.<br>Keywords with the AND/OR Boolean operators: (i) SARS-CoV-2, (ii) Coronavirus, (iii) COVID, (iv) COVID-19, (v) long-term, (vi) long covid, (vii) post acute covid, long haulers, (viii) erectile dysfunction, (ix) male sexual dysfunction.<br>Also looked through citations and references of relevant articles to retrieve additional resources. | To investigate and highlight the mechanisms through which long-term consequences of COVID-19 can affect erectile function, also discussing the current evidence concerning the epidemiology of long COVID and the potential treatments, wherever available. | Prevalence study, meta-analysis, systematic review. | July 2021 | July 2021 | None | Unclear (but appears to be hundreds of thousands of people in total). | Unclear | Results were limited to English, Italian and French. | 191 references listed. | Paper looks at more than Erectile Dysfunction - also looks at neuropsychiatric complications, respiratory complications, cardiovascular complications and endocrine complications. |
| **Castanares-Zapatero 2022[22]** | *Pathophysiology and mechanism of long COVID: a comprehensive review* | Belgium | - Studies putting forward hypotheses on the pathophysiological mechanisms that could contribute to persisting symptoms after COVID-19.<br>- Studies that included long COVID patients in their research | To conduct a comprehensive review to address the putative pathophysiology underlying the persisting symptoms of long COVID. | Case series, systematic reviews, cohort studies, and experimental studies. | February 2021 | 9th Aug 2021 | Belgian Health Care Knowledge Centre (KCE). | 14 exclusively included patients that were hospitalized during the acute infection phase.<br>11 included both hospitalized and non-hospitalized patients.<br>1 study exclusively comprised non- | Inclusion criteria formulated using the PEOD scheme:<br>- Studies putting forward hypotheses on the pathophysiological mechanisms that could contribute to persisting symptoms after COVID-19; | - Studies that exclusively focussed on acute mechanisms;<br>- Studies that put forward hypotheses on long-term putative consequences like neurodegenerative diseases or cancers;<br>- Case reports; | 98 | None. |

| | | | | | | | | | | | | | |
|---|---|---|---|---|---|---|---|---|---|---|---|---|---|
| | | | process.<br>A distinction was made between studies describing one or more hypotheses on the underlying pathophysiology of long COVID, and those analysing patient data. Article types were analysed separately and classified by organ system and symptoms. | | | | | | hospitalized patients. 5 studies did not mention the hospitalization status. | - Studies that included long COVID patients in their research process. The study designs were case series, systematic reviews, cohort studies, and experimental studies without any restriction concerning the included patient number. | - Articles were excluded if the content was essentially focussed on acute patho-physiological mechanisms underlying the acute infection phase;<br>- Languages were restricted to English, French, Dutch, and Spanish. | | |
| **Bergantini 2022[70]** | *Common Molecular Pathways Between Post-COVID19 Syndrome and Lung Fibrosis: A Scoping Review* | Italy | Terms: COVID-19 AND “lung fibrosis” OR “pulmonary fibrosis” OR “interstitial lung disease”, “pulmonary fibrosis and post-COVID19” OR “pulmonary fibrosis, post- COVID syndrome” | To carry out a systematic exploratory search of the literature (Scoping review) to identify and systematize the main pathogenetic mechanisms that are believed to be involved in this phenomenon, in order to highlight the same molecular aspect of the lung. | Included studies not limited to single design. | 19th March 2020 | 15th May 2021 | None | Unclear - appears to intend to include all patients with COVID19 infection who are still experiencing symptoms specifically relating to fibrosis. | Peer-reviewed empirical or perspective papers (including editorials or commentaries) with:<br>- Relevance to the study topic: Covid-19 disease or pandemic, fibrogenetic pathways, underlying pathogenetic mechanism, reciprocal influence between SARS-COV-2 and fibrotic lung disease;<br>- Type of journal: preferences for journals relating to the pneumology area with full text or abstract;<br>- Type of study: review, case report, case series, original article, letter to the editor. | - Did not satisfy the relevance to the topic of study;<br>- Did not adequately report objectives and conclusions;<br>- Did not carry full text. | 32 | None |
| **Meyer 2022[31]** | *Molecular Imaging Findings on Acute and Long-Term Effects of COVID-19 on the Brain: A Systematic Review* | Germany | Medline: (corona OR COVID OR SARS-CoV-2) AND (PET OR positron OR SPECT OR single-photon) AND (brain OR cerebral) | To provide a comprehensive, structured, and critical survey of actual knowledge on molecular imaging in neuropsychiatric COVID-19 manifestations. | Broad inclusion criteria: "peer-reviewed original studies and case series or case reports".<br>15 case reports/series and 10 original studies included. | 1st January 2019 | 31st December 2021 | Not reported | Mixture of hospitalised and non-hospitalised cases. | Studies using PET or SPECT to investigate central nervous system (CNS) manifestations of COVID-19. | Not stated. | 25 | None |
| **Akbarialiabad 2021[34]** | *Long COVID, a comprehensive systematic scoping review* | Iran | Keywords used: "long COVID" or "long haulers" or "post-acute COVID" or "chronic COVID syndrome" or "late sequela COVID" or "persistent COVID” (Google Translate used for non-English papers) | Identify what is known about Long Covid:<br>- diagnosis<br>- symptoms<br>- risk factors<br>-pathophysiology<br>- current recommended management | Included studies not limited to single design. | 20 January 2021 | 30 January 2021 | None | Unclear but appears to be all patients with ongoing symptoms following acute infection with COVID19. | - Re. long COVID, post-acute COVID, and long haulers of COVID-19<br>- Re. nomenclature, diagnosis criteria, pathophysiology, signs and symptoms, and managements<br>- All types of reports included: original | - Irrelevant to COVID-19<br>- Related to acute COVID-19<br>- Preprints<br>- Unavailable full texts | 120 | None |

| | | | | | | | | | | | | | |
|---|---|---|---|---|---|---|---|---|---|---|---|---|---|
| | | | | - Also identify gaps in knowledge. | | | | | | studies, reviews, editorials, viewpoints, guide-lines, letter to editors and commentaries<br>- Articles should have been published in a peer-reviewed journal or be an organizational report<br>- No language restriction | | | |
| **Anaya 2021[21]** | *Post-COVID syndrome. A case series and comprehensive review* | Colombia | Terms used: (“Post-COVID” OR “Long COVID”) AND (“COVID-19”). Articles in Spanish and English were included. | 1. To describe the clinical and serological characteristics (i.e. antibodies anti-SARS-CoV-2) of the first 100 consecutive patients attending a post-COVID Unit in Bogota, Colombia).<br>2. Conduct systematic review and meta-analysis. | Included publications not limited to single study design -30/40 studies in the systematic review were cohort studies, 5 cross-sectional, 3 case series, and 2 case-control studies. No clinical trials included in the systematic review and meta-analysis. | 18th March 2021 | 20th May 2021 | Grants from Universidad del Rosario. | Population unclear. Likely both hospitalised and community cases. | - Studies describing clinical manifestations after acute COVID-19<br>- Studies evaluating patients in clinical settings<br>- Case series, cross-sectional, case-control, cohort, clinical trial studies | Studies including data from national registries or unaudited databases. | 40 | There is a summary of characteristics from 100 (sequential) clinic patients AND a systematic review. Data extracted from the reviewed papers only. Only 7 of the 40 papers reported follow up timing (mean ~3months). |

| Systematic Reviews – Review 1, PART 2 | | | | |
|---|---|---|---|---|
| **Study ID** | **Title** | **Paper Symptoms and Clinical Features** | • **Paper Mechanisms** | **Paper Overall Conclusions** |
| **Salamanna 2021** [23] | *Post-COVID-19 Syndrome: The Persistent Symptoms at the Post-viral Stage of the Disease. A Systematic Review of the Current Data* | Persistent lung symptoms and dysfunctions by study design:<br>(a) Prospective cohort studies<br>- Reduced 6MWT, fatigue and dyspnea, 35.1% require home oxygen after hospital discharge<br>(b) Retrospective cohort studies<br>- Dyspnea, cough, fatigue, residual pulmonary disease, functional and QOL impairment, 65% with 6MWT below 80%; 52% free from exertional dyspnea according to mMRC scale<br>(c) Case report<br>- Abnormal airway function, cough, chest pain, chest tightness, shortness of breath, unstructured sleep apnea hypopnea syndrome, nocturnal sleep hypoxemia<br>Persistent neurological symptoms and olfactory dysfunctions by study design:<br>(a) Prospective cohort studies<br>- Altered sense of smell or taste/olfactory dysfunction, persistent headache, neurocognitive decline, psychiatric morbidity, poor QoL<br>(b) Retrospective cohort studies<br>- olfactory dysfunction; gustatory dysfunction, hearing loss, tinnitus, dizziness, spinning vertigo, dynamic imbalance, static imbalance, fatigue, memory/attention/cognitive deficits, sleep disorders, neurological abnormalities, hyposmia, postural tremor<br>(c) Case-control<br>- Resolution of anosmia or hyposmia in 85%<br>(d) Cross-sectional<br>- Myalgia, arthralgia, ADL restriction, sleeping troubles, nervousness and hopelessness, anorexia, chest pain, gastritis, cough, dyspnea, chemosensory dysfunction<br>(e) Case series<br>- Orthostatic intolerance syndromes: orthostatic hypotension; vasovagal syncope; postural orthostatic tachycardia syndrome, general cognitive decay, specific decline in attention, memory, language, praxis abilities<br>(f) Case report<br>- Deficits in working memory and digit span backwards with high average attentional skills, word finding difficulties, inefficient learning, decreased organisation, persistent psychotic symptoms, probable orthostatic hypoperfusion syndrome, painful small fiber neuropathy, intermittent neuropathic pain and paraesthesia in distal limbs, persistent anosmia, headaches, delirium and hallucinations<br>Widespread persistent symptoms by study design:<br>(a) Prospective cohort studies<br>- Breathlessness, fatigue, reduced HRQoL, anosmia/ageusia, asthenia, difficulty concentrating, fatigue, dyspnea, memory loss, confusion, headache, heart palpitations, chest pain, pain with deep breaths, dizziness, tachycardia, sleep diificulties/disturbance, PTSD, anxiety, depression, slow 4MGS, poor STS test result, muscle weakness, myalgia, arthralgia, lower scores on general health, physical health, mental health and social active role, impaired cognitive performance, reduced 6MWT distance<br>(b) Retrospective cohort studies<br>- Fatigue, dyspnea, joint pain, chest pain, asthenia, anosmia, aygeusia/dysgeusia, myalgia, ageusia, headache, microhaematuria, anxiety, low mood, sleep disturbance<br>(c) Case-control<br>- Chills, dyspnea; anosmia, dysgeusia, nausea, vomiting, cough, red eyes<br>(d) Cross-sectional<br>- Fatigue, muscle/joint pain, headache, insomnia, respiratory problems, palpitations, poor HRQoL, poor sleep quality, anxiety; dyspnea, joint pain, sense of fever, anorexia, diarrhoea, loss of taste and smell, cough, depression, non-return to full health<br>(e) Case series<br>- Dry cough, headache, severe sweating, shivering, loss of smell, mild on/off fever, diarrhoea, | • "Chun et al., evaluating 61 non-critical COVID-19 patients, highlighted higher levels of Lipocalin 2, suggesting that COVID-19 patients may have an ongoing neutrophil activation that could be amenable to targeted therapy."<br>• Potential mechanisms for lung symptoms and dysfunctions:<br>- Reduced DLCO<br>- Lung fibrotic-like change, residual ground-glass opacification, crazy-paving pattern, consolidation and linear opacities interstitial thickening, diffuse alveolar damage, extensive desquamation of proliferative type II alveolar epithelial cells, exudative monocytes and macrophages<br>- Pulmonary function abnormalities including reduced total lung capacity, reduced diffusion capacity<br>- Chest imaging abnormalities<br>- Iron deficiency, anemia<br>- Increased IL-6 and C-reactive protein<br>- Hyperferritinernia patients with severe lung pathologies<br>- LCN2, MMP-7, HGF higher in ICU subjects and inversely correlated with pulmonary function<br>- Cardiorespiratory cause of breathlessness, i.e. persistent parenchymal abnormality pulmonary embolism, cardiac complications<br>- Higher D-dimer level at admission predict impaired DLCO 3 months after<br>- Non-inflammatory tracheal hypersecretion<br>• Potential mechanisms for neurological symptoms and olfactory dysfunctions:<br>- Disruption to micro-structural and functional brain integrity<br>- General cognitive decay associated with ICU length of stay<br>- Hypometabolism of the olfactory/rectus gyrus in two cases<br>• Potential mechanisms for widespread symptoms:<br>- Persistent symptoms at 60-day follow-up significantly associated with age 40-60, hospital admission, and abnormal auscultation at symptom onset hyperfiltration had high blood pressure at diagnosis and still had prehypertension at 6-month follow-up<br>- MRI abnormalities in lung, heart, liver and kidney<br>- Persistent neuropsychiatric, pulmonary, metabolic, and coagulopathic phenotypes<br>- No association between fatigue and Covid severity<br>- Lymphopenia<br>- Elevated D-dimer, elevated C-reactive protein<br>- Abnormal x-rays of persistent infiltrate or atelectasis<br>- Extremely low outlier ratio of total protein, albumin, and globulin<br>- Persistent advanced atrioventricular block | Included studies vary by design and duration since COVID-19 onset, hence difficult to compare prevalence and longevity of symptoms/mechanisms.<br>Conjectures on mechanisms:<br>(1) *Most researchers and clinicians agree that the long-term COVID-19 symptoms are associated with the coronavirus ability to trigger a massive inflammatory response. Thus, it will be mandatory to analyse cytokine networks in patients who recover from COVID-19 to evaluate whether the , cytokine storm present during the disease persists and contributes to these long-term complications.*<br>(2) *Potential association between viral shedding and long-term COVID-19: some evidence that ongoing viral shedding in SARS-CoV-2 may be prolonged [beyond 12-20 days] in the feces compared to respiratory secretions The persistent fragments of viral genes, though not infectious, may still be triggering a violent immune overreaction that could explain the symptoms persistence in COVID-19-free patients.*<br>(3) *Even if the virus is cleared, the immune system could continue to be overactive or perturbed, analogous to the long-term debilitation after glandular fever.*<br>*"What emerges from this review is that the most common reported symptoms after COVID-19 are abnormal lung functions prevalently with persistent dyspnea, general neurological decay, smell and taste disturbances, and chronic fatigue."* |

| | | | | |
|---|---|---|---|---|
| | | weight loss<br>(f) Case report<br>- Chest pain, dyspnoea, fatigue, intercostal neuralgia, dysphonia, heart palpitations, chest pain, headache, difficulty concentrating, muscle weakness, dizziness, sore throat, hair shedding | | |
| **Garg 2021[2** | *The Conundrum of Long-COVID-19: A Narrative Review* | - Neuro-psychiatric: Flare-up of the neuropsychiatric symptoms or appearance of new psychiatric symptoms especially those who had critical illness or ICU admission, Impaired Cognition attention, Impaired concentration, Sleep disturbance<br>- Neurological: Brain Fog, Persistent loss of Smell/ Taste, Encephalopathy, Stroke<br>- Pulmonary: Breathlessness, Cough, Chest pain, ILD<br>- Psychiatric: Depression, Anxiety, PTSD, Social Isolation<br>CVD: Palpitations, Myocarditis/ Pericarditis, Heart Failure<br>- Cutaneous: Erythematous Rash, Urticarial Rash, COVID Toes<br>- General manifestations: Joint Pains, Peripheral Neuropathy, Peripheral Limb Ischemia | • Injury to the olfactory bulb demonstrated on MRI.<br>• The virus primarily affects the respiratory system, and after reaching the lung alveoli, it attaches and penetrates the host cell, particularly type II pneumocytes, and starts damaging the alveoli. It erodes into the capillary endothelium, causing endotheliitis and leads to the formation of microthrombi; then enters the blood vessels and may disseminate in the whole body.<br>• The virus also stimulates the inflammatory milieu in the body, causing significant release of cytokines and chemokines leading to cytokine storm in severe cases.<br>• Another important mechanism of extrapulmonary manifestations in COVID-19 is the formation of antigen-antibody complexes by the second or third week when the humoral immunity gets activated.<br>• Antigen-antibody reactions might be responsible for pulmonary fibrosis, and other long-term post-COVID consequences occurring in a few patients.<br>• The exact etiology of Long-COVID-19 is unclear, it appears secondary to endotheliopathy, hypoxemic injuries, antigen-antibody reactions, or aberrant immune response. | This review highlights the importance to be aware of its protean clinical manifestations, risk predictors, and holistic management strategies. Further research is needed focusing on cataloging of symptoms, longer-ranging observational studies, and clinical trials to evaluate the long-term consequences of COVID-19. As well as setting-up of dedicated, post-COVID care, multi-disciplinary clinics, and rehabilitation centers |
| **Joshee 2022[30]** | *Long-Term Effects of COVID-19* | Most common symptoms of long-term pulmonary sequelae include: fatigue, dyspnea, and/or cough | • Presence of autoreactive antibodies<br>• Inflammatory and metabolic changes to parenchyma, and supporting structures during initial infection<br>• Sequelae mediated by hospitalization interventions<br>• Immune-mediated damage to BBB & thromboembolism<br>• Viral mediated hypoxia and damage to PNS leading to neuropsychiatric, cognitive and peripheral nerve pathologies<br>• Inflammatory markers increase leakage and allow leukocyte infiltration and basement membrane modification<br>• Megakaryocytes in the parenchyma of alveolar tissue which may travel into the brain tissue due to endothelial disruption<br>• Hypoxia due to hypercoagulable state leading to HIF - I increase leading to increase in BBB permeability and prolonged cytokine release<br>• Viral mediated parenchyma damage<br>• Immune mediated microvascular damage leading to dyspnea, hypoxia, fatigue, ground glass opacities and pulmonary fibrosis<br>a) Virus binds to ACE2- cells release DAMPs/PAMPs<br>b) Macrophages release ILI and TNF-alpha - neutrophils attracted to side<br>c) Neutrophils release chemokines, increasing vascular permeability Differentiation of fibroblasts into myofibroblasts<br>d) Release of protein-rich exudate to interstitial space<br>e) Myofibroblasts release collagen, fibronectin and ECM in response to TGF-BETA<br>• Immune mediated endothelial dysfunction leading to venous, arterial, pulmonary thromboembolisms<br>• Innate immune system activation of type 1 interferon fuels pro-inflammatory and pro-coagulartion processes through endothelial cell dysfunction, endothelialitis, capillary leakage<br>Viral mediated parenchyma damage; immune mediated microvascular damage leading to AKI, glomerular and tubular diseases<br>a) ACE2 receptors in the proximal tubule apical brush border and podocytes<br>b) "second-hit phenomenon" where black patients with the high risk APOLI variant who also had COVID-19 are at an increased risk of collapsed glomerulopathy<br>c) Indirect mechanisms such as fluid imbalance, mechanical ventilation and organ crosstalk<br>• Viral mediated insulin decreases and resistance<br>• Immune-mediated endocrine parenchymal destruction leading to new-onset diabetes, | Recommendation of interdisciplinary monitoring required for all patients to detect post-acute covid-19 symptoms before long term systemic damage occurs. |

| | | | | |
|---|---|---|---|---|
| | | | worsening pre-existing diabetes, DKA, subacute thyroiditis, graves thyrotoxicosis<br>a) DKA development via downregulation of ACE2 receptors and damage of beta-islet during viral entry<br>b) ACE2 absence leads to unopposed angiotensin2 effects which impedes insulin secretion<br>c) Viral infections also induce insulin resistance to promote anti-viral effector CD8+ T-cells<br>d) Thyroid effects due to ACE/TMPSSR2 expression, secondary to HPA axs insult, or host inflammatory cytokine storm<br>• Immune-mediated myocardial and microvascular destruction leading to chest pain, palpitations, pericarditis, myocarditis, fibrosis, arrythmias/death<br>a) Endothelial cell disruption similar to pulmonary<br>b) Increased cardiometabolic demand leading to myocardial injury via hypoxia and overuse<br>c) Chronic myocarditis and IL6 leads to fibrofatty replacement<br>d) Fibrofatty replacement leads to reentrant arrythmias and sudden cardiac arrest and death<br>e) Medications also induce cardiotoxicity and electrolyte imbalances<br>• Viral mediated alterations in fecal microbiota - mechanism is unknown.<br>• Immune-mediated microvascular dysfunction leading to hair loss, skin rash, urticarial lesions, angioedema<br>a) Microvascular vasculitis from complement system activation, protein deposition in dermal capillaries, or direct viral effects<br>b) Hair loss due to COVID-19 has been attributed to telogen effluvium<br>c) Urticaria or angioedema may include a combination of post infectious immune dysregulation, adverse drug reactions, interruptions in urticaria therapy (omalizumab or oral antihistamine) or pandemic related stress. CD8+ T-cells ACE/TMPSSR2 inflammatory cytokine storm inflammatory Immune-mediated microvascular dysfunction; viral mediated effects unknown; stress | |
| **Tesarz 2022[24]** | *Pain, the brain, and SARS-CoV-2: evidence for pain-specific alterations in brain-related structure-function properties* | Persistent headache and musculoskeletal pain - time trend analyses indicate a biphasic course with a decrease in pain symptoms at the end of the first month after the initial COVID infection, a renewed increase after the end of the 2nd month, and with a second decrease more than six months after the acute disease phase. 15-20% of those with critical COVID subsequently develop chronic pain conditions. | Manifest structural damage in the context of acute COVID-19:<br>• Leukoencephalopathy and cerebral microbleeds are the most common MRI findings in the literature in patients with COVID-19 who have severe disease and a long duration of ventilation.<br>• Nonspecific white matter changes, preferentially localized in the frontal, parietal and temporal lobes and with a clustered localization in the subcortical white matter as well as in the corpus callosum and thalami<br>• Hypoxemia<br>• Abnormalities of the corpus callosum due to COVID-19 may affect brain networks crucial for pain, which could, in the long run, increase the vulnerability for pain problems.<br>• More widespread decrease in cerebral blood flow in the subcortical nuclei (mainly located in the striatum and amygdala) compared to less severe patients. These regions of the insula perform a variety of functions, ranging from sensory and affective processing to high-level cognition, including pain processing.<br>Histopathological brain studies of COVID-19 fatalities:<br>• Cascular changes<br>• Hyperemia of the meninges<br>• Perivascular inflammation in brain parenchyma with hypoxic neuronal damage<br>• Perivascular haemorrhage<br>• Gray matter disruption<br>• Blood-brain barrier damage.<br>• Brain changes caused by COVID-19 have been termed cerebral COVID-19 angiopathy with diffuse inflammation; such inflammatory changes concentrated in cerebral cortex, cerebellum, basal nuclei, and brainstem. Increased vascular permeability may be due to cytokine storm triggering endothelitis and general vasculopathic changes.<br>EEG findings:<br>• Abnormal background activity<br>• Slow frontal waves associated with metabolic and toxic encephalopathies<br>• No evidence of COVID-19 increasing neuronal excitability. No specific association with | The underlying pathophysiology for persistent pain following SARS-CoV-2 is still not understood, but central nervous processes seem to be of leading importance.<br>The infection may affect the central nervous pain system both via non-specific effects in the context of severe disease courses and the associated inflammatory processes and neurological complications and via specific effects, which may also occur in mild disease courses.<br>These lead to a hypometabolic metabolic state of the brain in specific brain regions, which include wide areas of the pain processing structures. The limbic structures, the frontal cortex, as well as thalamic and corpus callosum changes seem to be of critical importance here. Furthermore, the frontal control networks, which are also important for pain processing, as well as the dorsal attention and attentional networks, and their connectivity to the sensorimotor network appear to be affected. |

| | | | | |
|---|---|---|---|---|
| | | | persistent headache or other pain disorders identified.<br>Persistent hypometabolism in PET imaging:<br>• PET scans of 7 patients with COVID-19 related encephalopathy indicated impairment in cerebral glucose metabolism 6 months after disease<br>• PET analyses consistently show pattern of cerebral hypometabolism which appears to be associated with persistent pain<br>• Consistent pattern of hypometabolism after 6 months in frontal cortex, anterior cingulate, insula, and caudate nucleus (<br>• One study of two-thirds of patients with persistent pain identified four regional hypometabolic clusters: (i) frontal cluster - bilateral rectal/orbital gyrus, including the olfactory gyrus; (ii) right temporal cluster - right temporal lobe, including the amygdala and hippocampus, extending to the right thalamus; (iii) brainstem cluster - bilateral pons/medulla brainstemregion; (iv) cerebellar cluster - bilateral cerebellum.<br>• Persistent pain associated with lower metabolic values in all four clusters.<br>Cerebral hemodynamics and endothelial dysfunction:<br>• Increase in baseline cerebral blood velocity and a decrease in vasomotor reactivity in COVID-19 patients, which are hypothesised as consequences of endothelial dysfunction in the vascular structures of the central nervous system.<br>• Disturbances in vasomotor reactivity have been related to migraine-associated headaches.<br>Spatial-structural remodelling over the post-acute COVID:<br>• One study correlated post-COVID psychopathology with specific brain functional markers in COVID-19 survivors several weeks after illness resolution: correlation between severity of depressive psychopathology and decrease in gray matter volume in anterior cingulate cortex (ACC); between post-traumatic symptoms and decrease in gray matter volume in ACC and bilateral insular cortex; between severity of the initial inflammatory response in the acute stage and subsequent white matter microstructure changes and functional connectivity - increased connectivity within salience and sensorimotor networks has been associated with persistent physical pain.<br>• Lower the gray matter volume in the superior, medial, and middle frontal gyri, the higher the degree of cognitive impairment.<br>• One study found 2/3 of patients had multiple white matter lesions with a frontal and parietal distribution, particularly located in the cerebral hemispheres near the gray-white matter boundary;<br>• MRI abnormalities in patients who did not require intensive care<br>• Frontal networks represent a core region for long-term brain involvement in COVID-19 beyond the presence of specific (focal) damage associated with clinical manifestations (e.g., stroke)<br>• Study of COVID-19 patients with persistent nonspecific neurological symptoms (and control group) showed that mild-moderate severity patients had small, punctuate, newly formed hyperintense lesions on T2 and FLAIR sequences; number and severity of lesions correlated significantly with headache intensity<br>• Structural changes in the central olfactory system<br>• Patients with initially severe COVID-19 showed greater gray matter atrophy in limbic system components (left insula, left hippocampus, left superior temporal gyrus) than milder patients; structural changes correlated strongly with initial inflammatory marker levels | |
| **Houben 2022[29]** | *The Impact of COVID-19 Infection on Cognitive Function and the Implication for Rehabilitation: A Systematic Review and* | Paper focuses on cognitive assessments:<br>- Attention<br>- Executive Functions<br>- Fluency<br>- Processing Speed<br>- Verbal Memory<br>- Visuospatial Ability<br>- Working Memory<br>All studies report deficits in cognitive functions after COVID-19. | Evidence suggests a highly multifactorial component including:<br>• Direct infection by SARS-CoV-2<br>• Consequence of prolonged-time spent in intensive care units<br>• Persistent inflammation<br>• Brain hypoxia<br>• Ventilation mechanisms used<br>• Drugs<br>• Prior cognitive troubles<br>• Peripheral organ dysfunction. | People infected by SARS-CoV-2 show significant cognitive disorders independently from pathology and age.<br>No clear link between the severity of infection and degree of neurocognitive deficit.<br>Possible mechanisms include:<br>- Persistent inflammation/'Cytokine storm'<br>- Brain hypoxia<br>- Organ dysfunction |

| | | | | |
|---|---|---|---|---|
| | *Meta-Analysis* | | • Uncontrolled inflammatory response (cytokine storm) & disruption of the blood brain barrier may contribute to severity but not alone as cytokine storm only observable in severe cases & cognitive deficits seen in those with mild or severe COVID-19<br>• High levels of IL-6, IL-8, and TNF-found in serum<br>• Sustained inflammatory response could contribute to psychiatric sequelae after COVID-19<br>• As with other coronaviruses, SARS-CoV-2 shows a neurotropism. The virus could enter into neurons and glial cells with the SPIKE protein, which binds to ACE2 receptors (angiotensin-converting enzyme 2), which would result in neuronal death, causing cognitive deficits | - Neurotropism |
| **Pierce 2022[32]** | *Post-COVID-19 Syndrome* | - Top ten symptoms: Dyspnea, fatigue, loss of olfactory/gustatory function, tightness of the chest, chills or sweats, muscle or body aches, dry cough, sore throat, fever, headache or brain fog.<br>- Prevalence: dyspnea (70%), fatigue (60%).<br>- 47% of patients not on mechanical ventilation had significant persistent abnormal pulmonary function, whereas 90% of those who underwent invasive mechanical ventilation still had pulmonary problems at 100 days (Miwa et al., 2021) - possible indication of complication from therapeutic intervention.<br>- 15.1% of those discharged from hospitals were readmitted to hospital for post-COVID-19 complications at 60-day follow-up (Chopra et al., 2021). Of the post-COVID-19 patients, 32% still had persistent COVID-19 symptoms and 18.9% had new or deteriorating symptoms (Chopra et al., 2021).<br>- 70% of persons with post-COVID-19 symptoms who were physically fit before the illness now sedentary because of symptom severity (Carfi et al., 2020). | • Virus-specific pathophysiological variations:<br>- Direct viral injury. SARS-CoV-2 causes cellular damage by inflammatory cytokines, maladaptation of the ACE2 pathway, procoagulation, and other immune abnormalities. Viral toxicity contributes to sequelae seen in post-COVID-19 syndrome. The pathological condition resulting from SARS-CoV-2 or its sequelae leads to multiple organ dysfunction and infections. With the invasion of alveolar epithelial and endothelial cells by SARS-CoV-2, a cascade of neutrophils, monocytes, and immune cells leads to alveolar damage. In turn, numerous mechanisms and pathways are activated, causing sequelae such as heart failure, neuropsychiatric dysfunction, thromboembolism, renal injury, dermatological disorders, diabetic ketoacidosis, sensory defects, and irritable bowel syndrome.<br>• Oxidative stress:<br>SARS-CoV-2 can stimulate overactivation of the immune system. If SARS-CoV-2 causes severe pneumonia by infecting Type II pneumocytes, the mitochondria synthesising acetyl-CoA are damaged. The ability of mitochondria to produce antioxidants is also reduced. The latter induces imbalance between cellular reactive oxygen species (ROS) accumulation and antioxidant defences. It also leads to mitochondrial DNA mutations, injury to mitochondrial respiratory chain, modifications of membrane permeability, and activation of the mitochondrial defense systems. Moreover, oxygen supplementation can induce excess ROS generation in mitochondria. This damages the mitochondrial complexes and reduces oxidative phosphorylation leading to decreased adenosine triphosphate and increased cellular apoptosis. Mitochondrial ROS excess from hyperoxia can also affect immune and defense responses against virus. Administering antioxidants can decrease viral load and assist with recovery in post-COVID-19 syndrome: e.g., adding N-acetylcysteine and MitoQ to cells when peripheral blood monocytes were exposed to SARS-CoV-2 decreased viral replication.<br>• Immunologic abnormalities:<br>Acute COVID-19 patients with severe pneumonia or ARDS show a decrease in the lymphocyte counts and increased cytokines, particularly tumor necrosis factor and inflammatory cytokines, interleukins. Pulmonary injury or fibrosis due to cytokine storm induce dyspnea and chronic dry cough. Cytokine storm can also cause myocardial injury, leading to hypertrophy and fibrosis of the left ventricular wall. This hypertrophy decreases myocardial contractility and impairs cardiac function. The major cytokine for this damage is transforming growth factor-beta 1.<br>• Inflammatory damage:<br>In COVID-19 patients with both IFN-$\alpha\beta$ and IFN-gamma, there was inflammatory cell infiltration involving Fas-Fas ligand or TRAIL-death receptor 5 that cause airway and alveolar epithelial apoptosis. This inflammatory pathway leads to alveolar edema, hypoxia, and increased disease severity in other organs, such as heart failure. Multisystem inflammatory syndrome can cause many symptoms such as dyspnea, fatigue, pain, fever, nonspecific inflammation, and postviral arthritis.<br>• The multitude of symptoms experienced by persons with post-COVID-19 syndrome that persist for more than 4 weeks are often due to damage to the respiratory, cardiovascular, neurological, gastrointestinal, and other systems. | There is growing evidence that this is a complex and multifactorial syndrome involving virus-specific pathophysiological variations that affect many mechanisms but specifically oxidative stress, immune function, and inflammation.<br>Five studies found prolonged immunocompromise in people with post-COVID-19 symptoms.<br>Those with prolonged symptoms have augmented SARS-CoV-2-specific immune responses, particularly immune dysregulation.<br>Therapeutic treatments for persons who have post-COVID-19 syndrome should be based on the molecular and integrative physiological pathways that are damaged or dysfunctional. |
| **Piri 2021[35]** | *A systematic review on the recurrence of* | - Recurrence of SARS-CoV-2: 2.3% and 21.4% incidence in cohort studies; mean 20 days (range 1-140 days) after discharge; 7.3% severe or critical; 97.3% decreased or remained the same in severity compared to first infection. In most studies, the duration of being positive was shorter | Biomarkers in recurrent patients:<br>• Increased serum IL-6, increased lymphocyte counts and CT imaging features of lung consolidation during hospitalisation; concentrations of alanine aminotransferase (ALT) and | There is a relatively notable risk of disease recurrence in previously recovered patients, even those who are immunised against the virus . |

| | | | | |
|---|---|---|---|---|
| | *SARS-CoV-2 virus: frequency, risk factors, and possible explanations* | for recurrence than first infection.<br>- Potential reasons for recurrence: false-negative result at discharge; persistent shedding of virus; reinfection. Recurrence due to false-negative or reinfection cannot be seen as Long Covid symptom? - -- Proportions of recurrence cases due to above reasons unclear.<br>One found that all recurrent patients were non-severe cases, which suggests greater likelihood of virus persistence after discharge in non-severe cases due to their faster reach of the discharge criteria and shorter hospital stays, but two further studies found no association between disease severity and recurrence susceptibility. | aspartate aminotransferase (AST) noticeably increased in re-positive patients<br>• In one study, the possible association between the number of initial symptoms, fatigue, and creatine kinase levels with a re-positive RT-PCR test has been reported<br>Reasons for incomplete elimination of SARS-CoV-2 RNA:<br>• Shedding of the virus residue, which does not result in transmission<br>• Presence of latent SARS-CoV-2 infection within immune cells<br>• Multiplication of SARS-CoV-2 in the peripheral blood mononuclear cell (PBMC) through interaction with ACE2 receptor on the surface of human monocytes<br>• Entry of virus into T lymphocytes not accompanied by proliferation and the virus somehow settles in the cell - concentration of the viral genome in lymphocytes was higher than its concentration in plasma<br>• Because the epithelial cell half-life in the respiratory system is 3 months, the virus genome could be detected even after elimination during this time; (6) the mean time when the virus RNA PCR test of first SARS-Cov-2 infection became negative, it was 34 days in faecal specimens compared to 9 days in the respiratory samples, indicating that RNA could persist more in the gastrointestinal tract than respiratory tract and may be mistaken for reinfection.<br>Other:<br>• Another potential reason for reactivation of disease is the antibody dependent enhancement (ADE) phenomenon which can lead to more severe disease due to causing an unnecessary immune response<br>• Different studies observed no significant association between the recurrence susceptibility and the presence of these serum-specific antibodies. Hence, it could be speculated that antibody presence does not necessarily prevent disease recurrence<br>• Immunity failure may lead to prolonged viral shedding of the non-viable virus, replication of a viable virus, or reinfection. The absence of sufficient immunity in patients with re-positive SARS-CoV-2 test was observed. One paper stated that plasma obtained from COVID 19-positive individuals did not have high levels of neutralising antibodies but had receptor-binding domain-specific antibodies with strong antiviral activity. This had led to the hypothesis that high affinity versus low avidity could cause the virus to escape the immune system due to low levels of antibodies. However, over the time, the avidity of the antibodies increases, and diagnostic tests detect the viral remnants<br>• One paper showed that despite the usefulness of specific immunosuppressive drugs in the treatment of COVID-19, non-selective immunosuppressive drugs, such as prednisolone, may cause recurrence, even with first clinical improvement | In conclusion, although the underlying cause is not clear, recovered patients, even those with detectable anti-SARS-CoV-2 IgG may show disease recurrence. |
| **Willi 2021[25]** | *COVID-19 sequelae in adults aged less than 50 years: A systematic review* | Main long-term sequelae highlighted:<br>Persistent fatigue (39-73% of assessed persons); breathlessness (39-74%); decrease in quality of life (44-69%); impaired pulmonary function, abnormal CT findings including pulmonary fibrosis (39-83%); peri-/perimyo-/myocarditis (3-26%); changes in microstructural and functional brain integrity with persistent neurological symptoms (55%); increased incidence of psychiatric diagnoses (5.8% vs. 2.5-3.4% in controls); incomplete recovery of olfactory/gustatory dysfunction (33-36%), formerly employed participants have not returned to work, altered immune cell counts, thrombocytopenia, persistent lower limb weakness, subacute thyroiditis. | • Respiratory system: abnormal CT findings, impaired lung function observed, pulmonary fibrosis as radiological finding, hypoperfused lung volume.<br>• Cardiovascular & haematological system: peri-, myoperi-, and myocarditis in 3-26%, altered immune cell counts, thrombocytopenia.<br>• Neurological system & mental health: MRI findings of disruptions to microstructural and functional brain integrity.<br>• Endocrinological system: subacute thyroiditis.<br>• " SARS-CoV-2 can directly damage lung, heart, liver and kidney tissue, gastrointestinal mucosa, vascular endothelium, macrophages, T-lymphocytes and neurons. In some patients the infection leads to a massive release of cytokines, a cytokine storm, which indirectly results in extensive tissue damage."<br>• "A miscommunication in the inflammatory response pathways, especially cytokine networks, might be the underlying cause of chronic fatigue. It has been hypothesized that COVID-19 infection might lead to higher cancer risk through the activation of the MAPK and JAK-STAT pathway upon infection and the weakened immune system following a cytokine storm."<br>• Hypothesized role of SARS-CoV-2 in pancreatic damage and subsequent development of diabetes, in hypothalamic-pituitary-adrenal axis dysfunction and adrenal insufficiency and in hypothalamic-pituitary-thyroid axis dysfunction with thyroid damage, as seen in SARS-CoV infection. | Close attention should be paid to residual impairments in multi-organ function, especially persistent reduced lung function and carditis, and to mental health and neurological sequelae including post-viral fatigue syndrome.<br>Further research should include lung function tests and sensitive test batteries to detect long-lasting structural and functional damage to the cardiopulmonary and the neurological system.<br>Patients suffering from post-viral fatigue syndrome and mental health impairments could be followed-up routinely with questionnaires to monitor disease course.<br>In general, study test batteries to follow up on sequelae should be carefully designed to detect subtle, long-term sequelae. |

| | | | | |
|---|---|---|---|---|
| | | | • As ACE2 is highly expressed by the human testis, an infection might lead to testicular damage and associated male infertility. | |
| **Ramadan 2021[33]** | *Cardiac sequelae after coronavirus disease 2019 recovery: a systematic review* | - Increased T1 and T2 intensity<br>- Late gadolinium enhancement<br>- Pericardial effusion<br>- Decreased global longitudinal strain<br>- Decreased left ventricular ejection fraction<br>- Pericardial enhancement<br>- Elevated extracellular volume<br>- Pericardial effusion<br>- Global hypokinesis<br>- Left ventricular hypertrophy<br>- Diastolic dysfunction<br>- Pulmonary hypertension<br>- Reduced global longitudinal strain<br>- T-wave changes<br>- ST segment changes including elevation and depression<br>- Right bundle branch block<br>- Sinus tachycardia<br>- Elevated troponin<br>-Iincreased NT-pro-BNP levels<br>- Inflammation<br>- Chest pain<br>- Dyspnoea<br>- Palpitations<br>- Heart failure<br>- Myocardial infarction<br>- Stroke<br>- Arrhythmia<br>- Myocarditis<br>- Myopericarditis<br>- Pericarditis<br>- Hypertrophic cardiomyopathy<br>- Two-vessel coronary artery disease including left anterior descending artery occlusion in one patient and left anterior descending artery occlusion in another patient<br>Notes:<br>1. new-onset major cardiovascular adverse events totalling 66 per 1000 patient-years (compared with 12.3 per 1000 patient-years)<br>2. Re. CMR, 785 patients, "no study reported completely normal CMR results on all included patients". | • SARS-CoV-2 attaches to the angiotensin-converting enzyme 2 receptor, found on the surface of host cells, and highly expressed in the heart, kidneys, lungs and blood vessels. This could explain direct damage though cell invasion, translating into increased inflammation and coagulation, which could provide a pathophysiological basis for cardiac injury.<br>• "Short-term findings could point towards an active inflammatory process of the myocardium."<br>• "Diastolic dysfunction and pulmonary hypertension could result from direct viral injury and/or indirectly from chronic pulmonary disease and ongoing inflammation." | "Included studies demonstrate increased risk of clinical and subclinical cardiac sequelae in individuals who have recovered from COVID-19. Clinically, these patients seemed to be at a greater risk than controls who had never had COVID-19 for a range of cardiac diseases, including heart failure, myocardial infarction, myocarditis, pericarditis and arrythmia."<br>"Myocarditis was reported in groups with asymptomatic/mild COVID-and in healthy populations such as athletes, but without evidence of a greater arrythmia risk."<br><br>Potential mechanisms only briefly mentioned, primarily the virus attaching to ACE2 receptors causing direct damage, inflammation and coagulation. |
| **Michelen 2021[7]** | *Characterising long COVID: a living systematic review* | Most commonly reported symptoms (prevalence >=25%) were: weakness (41%); general malaise (33%); fatigue (31%); concentration impairment (26%); breathlessness (25%).<br><br>Hospitalised patients:<br>- Neurological and neuromuscular symptoms: headache; tremors; slowness of movement; lack of coordination; muscle atrophy; abnormal muscle tone; walking/gait abnormality; taste disturbance; smell disturbance; visual disturbance; decreased sensation or sensibility; tingling; trigeminal neuralgia; abnormal reflex status; other neurological diseases.<br>- Neurocognitive symptoms: memory impairment; concentration impairment; confusion; frontal release signs; other cognitive impairment.<br>- Psychological and social symptoms: anxiety; depression; sleep disorder; post-traumatic stress disorder; low mood; reduced quality of life; care dependency.<br>- Upper respiratory symptoms: sore throat; nasal congestion; voice change; other respiratory symptoms.<br>- Cardiopulmonary symptoms: breathlessness; chest pain; cough; excessive sputum; | • Lung CT scan data showed abnormal results, including consolidation, reticulation, residual ground glass opacity, interstitial thickening, and fibrotic changes.<br>• Pulmonary function test data found evidence of altered pulmonary function, most frequently significant reduction in carbon monoxide transfer factor.<br>• Low rates of hospital-associated venous thromboembolism at least 90 days post discharge.<br>• Decreased rate of kidney recovery for COVID-associated acute kidney injury (AKI) patients compared to non-COVID-associated AKI patients. | It is clear that long COVID affects different populations, with a wide range of symptomatology. Findings suggest this multiorgan syndrome is characterised by fatigue, weakness, malaise, breathlessness and concentration impairment, among other less frequent symptoms. Currently, the strength of the available evidence is limited and prone to bias. |

| | | | | |
|---|---|---|---|---|
| | | palpitations; flushing; newly diagnosed hypertension; other cardiovascular symptoms.<br>- Musculoskeletal symptoms: muscle pain; joint pain; impaired mobility.<br>- Gastrointestinal symptoms: nausea or vomiting; diarrhoea; loss of appetite; stomach/abdominal pain; other stomach/abdominal discomfort; weight loss; bloody stools.<br>- Systemic symptoms: fatigue; weakness; fever; sweat or night sweats; general malaise; dizziness.<br>- Other symptoms: skin rash; hair loss.<br><br>Non-hospitalised patients:<br>- Neurological and neuromuscular symptoms: headache; tremors; seizures/cramps; slowness of movement; lack of coordination; muscle atrophy; abnormal muscle tone; walking/gait abnormality; taste disturbance; smell disturbance; ear/hearing conditions; visual disturbance; decreased sensation or sensibility; tingling; abnormal reflex status; other neurological diseases.<br>- Neurocognitive symptoms: same as Hospitalised patients.<br>- Psychological and social symptoms: same as Hospitalised patients.<br>- Upper respiratory symptoms: sore throat; nasal congestion; other respiratory symptoms.<br>- Cardiopulmonary symptoms: breathlessness; chest pain; cough; excessive sputum; palpitations; other cardiovascular symptoms.<br>- Musculoskeletal symptoms: same as Hospitalised patients.<br>- Gastrointestinal symptoms: nausea or vomiting; diarrhoea; stomach/abdominal pain; weight loss.<br>- Systemic symptoms: fatigue; weakness; fever; sweat or night sweats; enlarged lymph nodes; dizziness.<br>- Other symptoms: skin rash; hair loss; conjunctivitis.<br><br>Statistically significant differences in prevalence rates between hospitalised and non-hospitalised patients (greater prevalence in the former) for fatigue, breathlessness, other respiratory symptoms, weight loss, tremors, memory impairment, and hair loss.<br><br>Significantly higher prevalence among non-hospitalised patients for chest pain and smell disturbance. | | |
| **Renaud-Charest 2021[26]** | *Onset and frequency of depression in post-COVID-19 syndrome: A systematic review* | - Frequency of depressive symptoms more than 12 weeks following SARS-CoV-2 infection ranged from 11 to 28%. When only considering clinically-significant depression and/or severe depressive symptoms, the reported rates ranged from 3 to 12%.<br>- Two studies found that patients with depression tended to perform worse on neurocognitive tests compared to those without depression.<br>Neurocognitive outcomes: selective attention and processing speed (symbol coding test); general cognitive function (MMSE); verbal memory (California Verbal Learning Test); visual reaction times (Test of Everyday Attention); executive abilities (Tower of London test); visuospatial abilities (Rey figure copy and recall). | • Neuropsychiatric symptoms (e.g., fatigue, depression) - suggest an effect of COVID-19 on the central nervous system (CNS) (e.g., neurotropism of SARS-CoV-2, hyperinflammatory state and hypercoagulability following infection, especially in severe cases.<br>• Main factors associated with depression frequency were sex, previous psychiatric history, psychopathology at one-month follow-up, and systemic inflammation during the acute phase, while age was only a potential factor, and severity of acute COVID-19 was not.<br>• Severity of depressive symptomatology in post-COVID-19 syndrome was proportional to systemic inflammation measured at baseline during acute infection. Systemic immune-inflammation index (SII) at baseline explained the variation of depression severity in models when assessing the influence of age, sex, and hospitalisation. The changes in SII also significantly predicted changes in depression scores. A decrease of SII between hospital admission and 3-month follow-up led to a decrease in depression severity in contrast to small changes of SII that led to persistent or worse depression scores.<br>• COVID-19 induces a hyperinflammatory state, which may cause persistent low-grade inflammation; more specifically, high levels of TNF-α, IFN-γ, IL-2, IL-4, IL-6, IL-10 and CRP in COVID-19 patients compared to controls, with IL-6 as the most frequently reported cytokine increased among COVID-19 patients. The positive association between depression and inflammation has also been characterised. Studies have reported elevated levels of pro-inflammatory cytokines (e.g., interleukin-6 (IL-6)) among patients suffering from mood symptoms and disorders.<br>• Literature indicates that pro-inflammatory cytokines moderate serotonin levels, hypothalamic-pituitary-adrenal (HPA) axis selfregulation, microglial cells, and neuroplasticity, leading to negative regulation of brain function.<br>• Three studies reported that severity of acute COVID-19 according to symptoms and treatment required did not have an influence on the frequency of depressive symptoms in | Included studies were highly heterogeneous with respect to mode of ascertainment, time of assessment, and location and age of patients.<br>The majority of studies did not include an unexposed control group.<br>Future research should endeavour to produce a standardized classification of post-COVID-19 syndrome, and include unexposed control groups.<br>It remains to be determined whether the high frequency of depression among individuals with post-COVID-19 syndrome<br>is a long-term consequence of the viral infection or a result of the social and/or economic outcomes of the pandemic.<br>This systematic review suggests a high frequency of clinically-significant depression and depressive symptoms associated with post-COVID-19 syndrome. Moderators include female sex, previous psychiatric history, and psychopathology at one-month follow-up. The severity of COVID-19 and cognitive impairment in the acute phase of the disease are not associated with worsening depressive symptoms in post-COVID-19 syndrome.<br>Nevertheless, it cannot be concluded that depression is more frequent in patients suffering from post-COVID-19 |

| | | | | |
|---|---|---|---|---|
| | | | post-COVID-19 syndrome. Notably, in one study, patients with mild COVID-19 disease reported greater frequency of depressive symptoms than patients with a critical form of the disease (22% vs 10%) 13.0 weeks after onset of symptoms.<br>• Non-intubated patients suffered more from depressive symptoms than intubated patients (21.7% vs. 18.0%) at a median of 125 days after hospital discharge. However, no statistical analysis was performed.<br>• One paper reported an inverse correlation between length of hospital stay and depression at three months post-discharge.<br>• Neurocognitive impairment in the acute phase of COVID-19 was not a moderator of depression. | syndrome than in the general population. |
| **Hussain 2022[20]** | *A systematic review of acute telogen effluvium, a harrowing post-COVID-19 manifestation* | - Mean duration from COVID-19 symptom onset to the appearance of acute telogen effluvium (TE) was 74 days.<br>- Positive telogen hair pull test in ~79% of patients.<br>- Trichoscopic findings most commonly included decreased hair density with the presence of empty follicles and short regrowing hair.<br>- In concordance with the findings of acute TE caused by other etiologies, post-COVID-19 acute TE also resolved in most patients.<br>- Patients should be advised to ensure a healthy diet and informed that it may take up to 18 months for hair thickness to return to baseline. | • Elevated levels of interleukin-6 seen in post-COVID patients. Interleukin-6 inhibits the elongation of the hair shaft by suppressing the proliferation of matrix cells in cultured hair follicles.<br>• Interferon is well-recognized to induce acute TE. Elevated levels of interferon are a well-known part of the body's antiviral response and ahve been documented in COVID-19 and other viral illnesses.<br>• Metalloproteinases 1 and 3 and interleukin-1β may inhibit growth of hair follicles.<br>• COVID-19 infection causes the activation of the coagulation cascade, which subsequently decreases the concentration of anticoagulation proteins due to diminished production and in-creased consumption. These factors may also result in microthrombi formation, which may occlude the blood supply of hair follicles.<br>• In COVID-19 patients, non-neutralizing virus-specific anti-bodies interact with Fcγ and/or complement receptors, facilitating the entry of the virus into the host cells. This may lead to direct viral damage to hair follicles. (this pathway to hair loss documented in 2014-2015 dengue epidemic).<br>• Medications used during COVID-19 treatment may contribute to acute TE. However, there is conflicting evidence regarding their potential pathways. Drug-induced acute TE in COVID-19 patients was unlikely due to the early onset of acute TE and the short duration of treatment for COVID-19. | Post-COVID,-19 acute TE occurred earlier than classic acute TE, but showed similar trichogram and trichoscopic findings, including reduced hair density, empty hair follicles, and/or short regrowing hair.<br>Proposed mechanisms:<br>1. Inflammation (raised IL-6);<br>2. Raised interferon levels;<br>3. Metalloproteinases 1 and 3 and interleukin-1β levels;<br>4. Microthrombi.<br>5. Interaction between non-neutralizing virus-specific anti-bodies with FcŒ≥ and/or complement receptors.<br>6. Medications used during Covid-19 infection. |
| **Ceban 2022[36]** | *Fatigue and cognitive impairment in Post-COVID-19 Syndrome: A systematic review and meta-analysis* | Primary outcomes:<br>- Fatigue<br>- Cognitive impairment<br>Secondary outcomes:<br>- Inflammatory parameters<br>- Functional outcomes/Quality of life | • Inflammatory parameters: 13/14 studies reported elevations in pro-inflammatory markers (pro-inflammatory cytokines, C-reactive peptide, D-dimer, and procalcitonin) in a subset of patients.<br>• 9 of those 14 studies reported pro-inflammatory markers and persistent fatigue and/or cognitive impairment.<br>• Several studies noted an association between elevation of measures of inflammation and PCS symptoms. | 1. Subset of individuals consistently exhibited markers of inflammation following resolution of acute COVID-19, suggesting hyperinflammation is a cause of fatigue and/or cognitive impairment in PCS.<br>2. Authors also reference other studies NOT INCLUDED IN MAIN REVIEW to suggest other pathophysiology:<br>- endothelial dysfunction<br>- hyperinflammation<br>- autoimmunity<br>- latent viral reactivation - multi-organ pathology<br>- autonomic nervous system dysfunction - pro-inflammatory cytokines, mood symptoms, and cognitive decline. |
| **Sansone 2022[27]** | *The Sexual Long COVID (SLC): Erectile Dysfunction as a Biomarker of Systemic Complications for COVID-19 Long Haulers* | - Neuropsychiatric Complications of Long COVID involvement of the neurological system at many levels, from cognition to cerebrovascular events to sensory dysfunction. Anosmia and ageusia.<br>- Fatigue, headache and "brain fog"<br>- Respiratory complications - dry cough, dyspnea, hypoxemia, and abnormal imaging results.<br>- Cardiovascular complications, including tachycardia, chest pain, dyspnea, arrhythmias, POTS thrombotic events, emboli, myocarditis.<br>Endocrine complications, including thyrotoxicosis, pituitary apoplexy, onset of type 1 and type 2 diabetes, reduced testosterone levels.<br>Relevance for erectile function:<br>- The cognitive defects and sleep disorders featured in long COVID contribute to poorer sexual | • The chemokine profile featured in the "cytokinestorm" can promote neuroinflammation, endothelial dysfunction in brain vessels, vascular thrombosis and local hypoxia, and can also contribute to the onset of potential long-term neurological defects. As SARS-CoV-2 has been identified in the cerebrospinal fluid, encephalitis following direct viral neuronal damage has been included among the complications of COVID-19. SARS-CoV-2 has also been shown to infect brain endothelial cells, resulting in microvascular pathology occurring in brain vessels which can also influence the blood-brain barrier.<br>• Several central and peripheral organic factors considered, including systemic low-grade inflammation, impaired cerebrospinal fluid drainage and muscle damage... At present, exact mechanisms leading to the development of brain fog are not fully understood; however they are likely mild complications of COVID-related encephalitis, as also shown by the finding of | "Erectile disorders might therefore be a clinically tangible warning of the complex web of underlying vascular, endothelial, metabolic, neuropsychiatric and pulmonary risk factors."<br>Proposed mechanisms for Long Covid symptoms include:<br>- Inflammation;<br>- Endothelial dysfunction;<br>- Thrombosis;<br>- Hypoxia;<br>- Neuronal damage; |

| | | | | |
|---|---|---|---|---|
| | | health.<br>- Likewise, chronic fatigue has been associated with declining sexual functioning.<br>- Anxiety, depression and PTSD have all been long considered risk factors for the development of sexual dysfunctions, the relation between sexual and mental health is bidirectional.<br>- Anosmia and ageusia: both conditions can negatively affect sexual behavior, owing to the "emotional" involvement of smell and taste in arousal and intimacy and might persist for several months, blunting sexual interest.<br>- Dyspnea and shortness of breath are common long-term complaints for COVID-19 patients. Sexual dysfunctions are known to occur in all conditions of impaired oxygen availability.<br>- Erectile function is strictly dependent on the integrity of the endothelium.<br>- As sexual activity can be considered a mild form of physical exercise, accounting for 3-5 metabolic equivalents, it can become somewhat hazardous in patients with myocarditis or with a sudden onset of chest pain - even more in case of pulmonary dysfunction. Likewise, patients with POTS might experience several debilitating symptoms, such as shortness of breath, sweating, and nausea, when changing position during sexual intercourse.<br>- Several drugs used in the clinical management of COVID-19 long term complications, including beta-blockers and diuretics, might have negative effects on erectile function. | abnormal neurologic exam, possibly associated with mast cells activation and release of proinflammatory cytokines.<br>• The inflammatory response (particularly interleukin-6, which is among the main drivers of inflammation for COVID-19) and the endothelial dysfunction occurring in the small vessels of the pulmonary circulation, induce pulmonary dysfunction, as also proven by the high prevalence of residual lung abnormalities at computed tomography (CT), such as ground glass opacity and signs of pulmonary fibrosis, occurring in 44.1% and 33.9% patients at three months. Oxygen saturation decreases following pulmonary fibrosis, as also occurring in COVID-19 patients due to the combination of anaemic and hypoxic hypoxia. As oxygen is a necessary substrate for the production of nitric oxide (NO), pulmonary fibrosis negatively affects the erectile function.<br>• First line of treatment for ED involves the use of phosphodiesterase type V inhibitors (PDE5i), vasoactive agents which act downstream of NO synthesis; such drugs also improve pulmonary vasodilation, allowing for an increased alveolar gas exchange. Additional benefits: sildenafil, the first and most widely known PDE5i, has anti-aggregation properties and inhibits neointimal hyperplasia through the cGMP-dependent protein kinase (cGK) pathway, thus preventing further vascular injury and thrombosis, and has anti-inflammatory, anti-apoptotic and anti-oxidant properties which could improve inflammatory status in the lungs. Therefore, PDE5i can potentially be beneficial for both erectile and pulmonary function in long COVID patients.<br>• As endothelial cells express ACE2 and TMPRSS2, SARS-CoV-2 can invade them, resulting in a diffuse form of endotheliitis, which has been considered one of the main drivers of the microcirculatory dysfunction occurring in COVID-19. Unsurprisingly, patients with more severe forms of COVID-related vascular damage, including acute thrombotic events and pulmonary embolism, often also show more severe endothelial dysfunction. The persistent endothelial dysfunction is, however, part of a more complex phenotype, supported by the unfavourable immune response and chemokine profile: several mechanisms have been considered, including coagulopathy, chronic inflammation of cardiomyocytes, myocardial ischemia and local hypoxia, ultimately resulting in fibrotic changes in the heart.<br>• Endothelial dysfunction is likely to have negative repercussions on erection, which undoubtedly result in impaired sexual health and satisfaction. Such effects have been shown to act regardless of anxiety and depression, suggesting that endothelial function might actually be an independent risk factor for the development of ED in affected patients.<br>• Additional support in these regards comes from the presence of SARS-CoV-2 particles and the reduced expression of endothelial NO synthase (eNOS) expression in the endothelium of COVID-19 patients. The chemokine profile described in COVID-19 can also contribute to the progression to more severe forms of erectile dysfunction.<br>• ACE2 is highly expressed by several cell types, including many highly relevant for the endocrine system such as pancreatic b-cells, hypothalamic and pituitary cells, and testicular Leydig and Sertoli cells. Viral direct damage can therefore trigger endocrine complications. Pituitary apoplexy - possibly secondary to endothelial dysfunction, coagulopathy and increased blood flow due to the underlying inflammatory condition.<br>• Type I and Type 2 diabetes - onset of type I diabetes mellitus in predisposed individuals following autoimmune reaction towards the b-cells, as well as to accelerated development of type II diabetes mellitus due to pancreatic inflammation, islet remodelling and progressive b-cell dysfunction. | - Impaired cerebrospinal fluid drainage;<br>- Muscle damage;<br>- Mast cell activation;<br>- Lung abnormalities from Covid-19 damage;<br>- Viral damage of cells in the endocrine system;<br>- Immune response;<br>- Autonomic nervous system involvement. |
| **Castanares-Zapatero 2022[22]** | *Pathophysiology and mechanism of long COVID: a comprehensive review* | - Cognitive and mental health disorders;<br>- Pain<br>- Headache<br>- Fatigue<br>- Anosmia/Agueusia<br>- Neuropathy<br>- Dyspnoea<br>- Chest pain | • Hypometabolic activity in various cerebral zones<br>• Reduced activity of the GABA inhibition<br>• Neuro-inflammation and brain microstructural modifications<br>• Micro-structural, volumetric and vascularization disorders<br>• Structural lesions in the olfactory and taste system identified at imaging and histology<br>• Injury in olfactory neuronal pathways<br>• Persistent inflammation of the neuroepithelium and with SARS-CoV-2 RNA identification<br>• Invasion and replication of SARS-CoV-2 in taste buds type II cells | Various mechanisms have been identified as being potentially involved in symptoms of Long COVID, including:<br>- Neuro-inflammation<br>- Activation of coagulation<br>- Autoimmunity<br>- Metabolic brain disorders<br>- Residual virus particle, persistent smoldering |

| | | | | |
|---|---|---|---|---|
| | | - Cough<br>- GI Tract<br>- Skin disorders | • Persistent vascular inflammation<br>• Macrovascular vascular inflammation<br>• Microvascular inflammation: increased level of cytokines, circulating endothelial cells, coagulation activation microvascular retinal impairment (at autopsy, evidence of endothelial cells and<br>cardiomyocytes viral invasion with signs of structural alterations)<br>• Auto-immunity: auto-antibodies able to modulate the cardiac frequency and vascular tone (acting as receptor agonists on the b2-adrenoceptor, the a1- adrenoceptor, angiotensin II AT1-receptor, angiotensin 1,7 and endothelin receptors)<br>• Persistent alteration of coagulation (sustained increased of Ddimer levels)<br>• Persistent inflammation and dysregulated host response of lung repair<br>• Increased plasma biomarkers of lung inflammation and fibrosis (Lipocalin 2, Matrix metalloproteinase-7, Hepatocyte growth factor)<br>• Persisting inflammation in lungs, mediastinal lymph nodes, spleen, and liver<br>• Involvement of iron homeostasis disturbances in end-organ damage<br>• Gut microbiota modifications after recovery<br>• Decreases gut commensals with known immunomodulatory potential<br>• Perturbed composition of microbiota correlated inflammation biomarkers<br>• Persistent immune inflammatory response impairing organ functioning<br>• Remaining inflammation in blood samples analysis, long-lasting phenotypic and functional disorders of lymphocytes, decreased amounts of dendritic cells and persisting alterations of activation markers<br>• Signs of mild organ impairment at magnetic resonance imaging and FDG PET/CT<br>• Autoimmunity: auto-antibodies against the nociceptive receptors, immunomodulatory proteins (including cytokines, chemokines, complement components, and cell-surface proteins) and tissue components<br>• Persistence of the SARS-CoV-2 nucleic acids in tissues<br>• Multisystem Inflammatory syndrome in children (MIS-C)<br>• At biopsy, presence of lymphocytic or neutrophilic infiltrates, endothelitis, microangiopathy, and microthrombosis | infection<br>- Direct or indirect nervous system damage during the acute phase<br>- Activation of nerves<br>- Glymphatic-lymphatic system congestion<br>- Bioenergetic disorders in muscles due to mitochondria dysfunction<br>- Olfactory dysfunction due to viral invasion of olfactory mucosa<br>- Endothelial dysfunction<br>- Antiphospholipid antibodies<br>- Damages of the autonomic nervous system<br>- Lung fibrosis, pulmonary vasculature damages<br>- Chronic dysregulated immune system activation<br>- Mast cell activation syndrome<br>- Disruption of myocytes and fibroblast activation<br>- Alteration of microcirculation in bones, autoimmunity and NETs activation in joints<br>- Post-infection gastro-intestinal dysfunction, microbiota alterations, hepato-biliary damage, renal cell viral invasion<br>- Direct damage on the thyroid gland, subacute thyroiditis, low-T3 syndrome<br>- Uncontrolled T-cell immune response<br>- Molecular mimicry between antigens |

| | | | | |
|---|---|---|---|---|
| **Bergantini 2022[70]** | *Common Molecular Pathways Between Post-COVID19 Syndrome and Lung Fibrosis: A Scoping Review* | Lung fibrosis. | • Molecular mechanisms of PF secondary to COVID-19 are mainly related to the TNF signaling pathway, the cytokine-cytokine receptor interaction pathway and the NF-kB signaling pathway.<br>• Among cytokines, interleukin 6 (IL-6), TNF and IL-1β have been identified as key targets associated<br>with PF secondary to COVID-19.<br>• Oulmonary fibrosis tissue from IPF patients have shown an increased expression of ACE2 in fibroblasts, make fibrotic patients more susceptible to virus entrance. | A thorough discussion of fibrosis and its general pathology with logical reasoning for similar pathways to fibrosis following COVID19.<br>ACE2 seems to play a role because of its widespread systemic nature.<br>Several gene pathways seem to be activated by SARS-CoV-2 leading to increase of cytokines and epitheleal/endothelial activity - also known to influence fibrosis.<br>Broad message is that cytokine storm leads to dysregulated repair leading to fibrogenesis (the two studies cited for this don't appear in the review though and are authored by the first author of this review).<br>Specific substances that can trigger fibrosis/fibrosis-like lung injury include TGF-Œ≤, IL-4, IL-6 and IL-13.<br>Fibrosis is a normal healing process but in the case of repeated or chronic lung damage, fibrosis becomes aberrant wound healing due to the dysregulation of the fibroblasts and the extensive deposition of collagen and elastin.<br>Risk factors for extensive or prolonged pulmonary fibrosis in long COVID include shortened telomere length (although its unclear if this might not just be an age-related factor as telomeres shorten with age). |
| **Meyer 2022[31]** | *Molecular Imaging Findings on Acute and Long-Term Effects of COVID-19 on the Brain: A Systematic Review* | - Focal symptoms: olfactory dysfunction being most frequent<br>- Encephalopathy (around 70% of hospitalised patients) - characterised by cognitive impairment and other neurologic signs (e.g., hemiparesis, ataxia, apraxia, aphasia, myoclonus, and seizures)<br>- Post-COVID-19 syndrome - mostly memory or cognitive complaints, fatigue, and insomnia | (Data from 1 case report and 4 cohort studies of adults and children.)<br>• Long-COVID patients showed extensive areas of hypometabolism including the orbitofrontal cortex bilaterally, the medical temporal lobes bilaterally, the right thalamus (adults only), and the brain stem and cerebellum.<br>• Proposed mechanisms: For adults, neurotropism of virus through the olfactory bulb, with extension of impairment to the limbic and other regions.<br>• PET scan data conflicting: One study showed long-COVID patients exhibited hypometabolism of the right parahippocampal gyrus and thalamus relative to melanoma patients. Another study, scans found no significant regional abnormality relative to controls. | In patients with a high level of self-reported complaints, some authors have found extensive areas of limbic and subcortical hypometabolism, whereas others found no metabolic alterations on PET and only minor cognitive impairments on neuropsychologic assessment.<br>Given the complexity, costs, and radiation exposure, cohorts in PET studies are often rather small, which limits their statistical power.<br>The contribution of 18F-FDG PET to understanding and diagnosing a post-COVID-19 syndrome dominated by fatigue is limited. |
| **Akbarialiabad 2021[34]** | *Long COVID, a comprehensive systematic scoping review* | Thirteen (10.8%) articles evaluated pathophysiology including:<br>- 8 neurologic<br>- 2 cardiovascular<br>- 1 fatigue<br>- 1 MIS in paediatric<br>- 1 pathophysiology of multiple systems | • ACE2 receptor expressed in many tissues<br>• Oxidative stress and inflammation leads to weak immunologic response and incomplete virus eradication<br>• Virus residuals and antigen remnants cause ongoing inflammatory response /vicious cycle<br>• Persistence of viremia and insufficient antibody generation<br>• Psychological factors e.g. PTSD may contribute to development of Long COVID (single patient case report)<br>• Genetic profile primarily related to the immune system, such as HLA<br>• RNA of SARS-COV-2 may remain in the CNS after acute phase and may result in neuronal loss<br>• Systemic inflammation causes generalized endothelitis and disruption of blood-brain barrier<br>• Systemic hyper-inflammation is a leading cause of neurodegeneration and cognitive decline<br>• Direct invasion of virus responsible for persistent neuropsychiatric features of SARS-COV-2<br>• Dysregulated immunologic response and virus-induced cytokine storm - production of pro-inflammatory cytokines such as IL-7 and IFNγ can result in post-stroke depression<br>• COVID-19 patients have a higher level of NLRP3 inflammasome activation that in combination with interleukin-18 and interleukin-1β have been shown to adversely impact cerebral function<br>• NLRP3 inflammasome-mediated systemic inflammation can lead to pathological | Few suggested mechanisms are from original studies. Those that are, are small and range from single case reports to ~100 patients.<br>Many of the 'results' presented in this paper are taken from opinion articles rather than from articles identified to be included in the review.<br>Some of the mechanisms relate to acute Covid symptoms and also speculate on findings relating to other conditions (e.g. stroke). |

| | | | | |
|---|---|---|---|---|
| | | | • accumulation of peptides/proteins such as fibrillar amyloid-β resulting in the induction and aggravation of neurodegenerative illnesses such as Alzheimer's disease<br>• SARS-COV-2 infection may impair cognitive function via selective targeting of the mitochondria of the neurons<br>• Tachycardia, palpitation, orthostatic intolerance, breathlessness, chest pain as the consequences of autonomic nervous system instability caused by deconditioning, hypovolemia or immune- or virus-mediated autonomic nervous system destruction<br>• Activated T-cells and T-cells-specific for unrelated antigens were pre-sent, suggesting "a significant amount of by stander activation" which might contribute to recurring anti-bacterial-resistant infections<br>• High levels of biomarkers related to innate anti-inflammatory and stress response were present | |
| **Anaya 2021[21]** | *Post-COVID syndrome. A case series and comprehensive review* | - Fatigue/muscle weakness (25 papers)<br>- Dyspnea (23 papers)<br>- Pain/discomfort (9 papers plus 17 specific to headache and 15 specific to chest/pleuritic pain)<br>- Anxiety/depression (12 papers)<br>- Impaired concentration (19 papers) | • Analysis (a K-means clustering analysis based on the algorithm of Hartigan and Wong on weighted COMPASS 31 domains was conducted) suggests autoimmune response related to the following post-covid symptoms: Impaired visual acuity/blurry vision, depression, chills, weakness, myalgia, back pain, heavy arms/legs, inability to walk, fatigue, body pain, arthralgia, palpitations, xerostomia, chest tightness, tachycardia, edema, parathesia, attention disorders, seizures, headache, dizziness, vision disorders, eye pain. | Mechanisms behind autonomic dysfunction are not clear. However, autoimmune phenomena could be associated with its appearance. Production of autoantibodies against $\alpha 1$, β1 and β2 receptors, angiotensin II receptor type 1, opioids 1, acetylcholine, M2 and M4S receptors are associated with development of POTS. This immune mechanism, secondary to SARS-CoV2, could be triggered through molecular mimicry between antigens of autonomic nerve fibers, autonomic ganglia, and SARS-CoV2 antigens, as described in some autoimmune conditions. |
