# Supplementary material for: Pathophysiological Mechanisms in Long COVID: A Mixed Method Systematic Review
Source: Int J Environ Res Public Health. 2024 Apr 12;21(4):473. doi: 10.3390/ijerph21040473 (PMC11050596; doi:10.3390/ijerph21040473)
Supplement: Supplementary file 1 [file ijerph-21-00473-s001.zip › Table S2_ Summary of findings in Review 2. Draft 2. NDB edits 10.4.24.pdf]

Table S2. Summary of findings in Review 2.

Individual Papers - Review 2, Part 1											
**Study ID**	**Title**	**Country in which Study Conducted**	**Aim of Study**	**Study Design**	**Start Date**	**End Date**	**Study Funding Sources**	**Population Description**	**Inclusion Criteria**	**Exclusion Criteria**	**Method of Recruitment of Participants**
**Holmes 2021[52]**	*Incomplete Systemic Recovery and Metabolic Phenoreversion in Post-Acute-Phase Nonhospitalized COVID-19 Patients: Implications for Assessment of Post-Acute COVID-19 Syndrome*	Australia	To develop the basis of an objective metabolic framework for measuring systemic recovery in COVID-19 patients using a range of metabolic technologies and biomarkers.	Cohort study with control group.	Not stated	Not stated	Multiple funding sources including government bodies and charitable organisations.	Three cohorts:<br>1) healthy control group (n = 41);<br>2) hospitalized patient group (n = 18 with multiple time- points) sampled during the acute infection phase;<br>3) recovery cohort consisting of a non-hospitalized group sampled 3 months post the acute phase of the disease (n = 27) with acute- phase symptoms and symptoms 6 months post COVID infection.	Other than defining the 3 main cohorts, there is no information of how or when participants were selected.	As above	Not clear
**Weinstock 2021[84]**	*Mast cell activation symptoms are prevalent in Long-COVID*	United States	To determine the prevalence and severity of mast cell activation (MCA) symptoms in Long COVID patients to test the hypothesis that hyper-inflammation during COVID-19 and LC are mediated by MCA.<br><br>Whether prevalence and severity of MCA symptoms in LC patients were: (1) similar to healthy controls at pre-COVID-19 status; (2) significantly higher after COVID-19; and (3) reaching a level that resembled MCAS patients.	Case control study	October 2020	May 2021	Missouri Baptist Healthcare Foundation	1) Long Covid patients: infection confirmed by testing or by physician;<br>2) General population controls: employees and spouses from Missouri Baptist Medical Center and offices of the lead investigator;<br>3) MCAS patients: diagnosed in last two years by lead investigator. (Criteria for diagnosing MCAS: typical symptoms of MCA in 5+ organ systems; 1+ elevated mast cell mediator(s); clinical improvement with MC-directed therapy. Mast cell mediator release syndrome (MCMRS) score used to confirm diagnosis and assess severity of MCA; score >=14 consistent with MCMRS.)	Controls and MCAS patients without history of symptoms of acute COVID-19 infection.	- Under 18 years old<br>- Pregnancy	Recruited LC-focused Facebook support groups
**Files 2021[38]**	*Duration of post-COVID-19 symptoms is associated with sustained SARS-CoV-2-specific immune responses*	United States	Researchers comprehensively profiled longitudinal samples from convalescent patients to assess potential immune differences between individuals experiencing prolonged symptom duration and those with complete recovery.	Cohort study	Not stated	Not stated	Intramural	Unclear, but probably patients being tested at single hospital laboratory.	PCR positive, pre-defined set of LC symptoms at 30 days.	Not clear	Clinic patients
**Glynne 2022[65]**	*Long-COVID following mild SARS CoV-2 infection: characteristic T cell alterations and response to antihistamines*	UK	To survey T-cell populations in patients recovered from mild COVID-19, comparing those with long-COVID and asymptomatic individuals, and to analyse these data in light of symptoms and response to HRA.	Cohort study with control group	November 2020	April 2021	Philanthropic donations from The Dominvs Group and Sir Peter Wood	1) 49 with diagnosis of long-COVID were recruited. 25 had either PCR or serological evidence for COVID-19. The remainder had suffered acute illnesses at start of pandemic when PCR testing was not widely available in the UK, and had not subsequently returned positive serology tests.	- 87 to 408 days post mild COVID-19;<br>- None had sought treatment for acute COVID-19.	None had previously sought medical attention or “treatment” for acute COVID-19, none had a history of autoimmunity, and none had received immunomodulatory medications.	Voluntary

								2) 16 individuals known to have had acute COVID infection but who had recovered rapidly and uneventfully to serve as asymptomatic controls.			
**Apple 2022[37]**	*Risk factors and abnormal cerebrospinal fluid associate with cognitive symptoms after mild COVID-19*	United States	To identify salient clinical factors associated with cognitive PASC after mild COVID-19 that may inform pathogenesis.	Referred by clinician or self-referred	Not stated	Not stated	Dr. Pleasure & Dr. Hellmuth: NIH grants	1) PASC group: 22 adults reporting cognitive post-acute sequelae of SARS-CoV-2 (PASC);<br>2) Controls: 10 not reporting cognitive symptoms after mild SARS-CoV-2 infection.	Participants in Coronavirus Neurocognitive Study, a sub-study of the Long-term Impact of Infection with Novel Coronavirus (LIINC) study enrolled 14 days+ from symptom onset with confirmed SARS-CoV-2 by nucleic acid amplification test. Not hospitalised with COVID-19 and no significant medical complications (e.g., thrombotic events).	- Inability to complete evaluations in English;<br>- history of serious or untreated medical or psychiatric condition(s) that may confound cognitive issues;<br>- active substance use disorder;<br>- daily recreational substance use.	Subgroup within a survey which enrolled individuals referred by clinicians or self-referred.
**Fogarty 2021[51**	*Persistent endotheliopathy in the pathogenesis of long COVID syndrome*	Ireland, Germany, UK, Australia	To examine the relationship between Long Covid clinical features, clotting factors and endothelial function.	Case control study	May 2020	September 2020	Health Research Board COVID-19 Rapid Response award National Children Research Centre, Grant/Award Wellcome Trust/HRB ICAT Fellowship Programme Grant/Award 3M Foundation	Consecutive adult patients from the St James' Hospital COVID review clinic and a control group of asymptomatic non-hospitalised patients.	Patients attending the post-COVID-19 review clinic.	None	Clinic patients
**Ferrando 2022[68]**	*Neuropsychological, Medical, and Psychiatric Findings After Recovery From Acute COVID-19: A Cross-sectional Study*	United States	To investigate longer term neuropsychiatric sequelae of COVID-19 by assessing individuals recovered from an acute COVID-19 illness with NP, psychiatric, medical, and sociodemographic instruments.	Case control study	Not stated	Not stated	Intramural	1) Long Covid: Mean age - 48.1; Mean number of comorbidities - 1.9; 19% hospitalised<br>Mean days since diagnosis - 250; Mean acute COVID severity score -18.9;<br>2) Controls: Mean age - 33.7; Mean number of comorbidities - 1.0; 4% hospitalised, mean days since diagnosis – 172; Mean acute COVID severity score - 13.7<br>Note: Author-stated limitation - "The study did not include a COVID-19-negative comparison group matched for age, medical, and other comorbidities."	- Age >=20 years;<br>- Documented positive COVID-19 nasopharyngeal test or positive antibody test before vaccination;<br>- Recovered from acute COVID-19 infection as per CDC recommendations;<br>- Completed minimum 8th grade education;<br>- Fluent in English;<br>- Capable of informed consent.	- Prior diagnosis of a major neuro-cognitive disorder;<br>- traumatic brain injury with loss of consciousness;<br>- uncorrected visual/hearing deficits;<br>- intellectual disability;<br>- unstable psychiatric symptoms.	Voluntary (invited by adverts)
**Lehmann 2022[79]**	*Comparison of pulmonary function test, diffusion capacity, blood gas analysis and CT scan in patients with and without persistent respiratory symptoms following COVID-19*	Austria	To assess the presence of abnormal pulmonary function tests, radiological and laboratory findings in patients with and without ongoing respiratory symptoms following COVID-19.	Retrospective cohort study	June 2020	Not stated	Medizinisch-Wissenschaftlichen Fonds des Bürgermeisters der Bundeshauptstadt Wien	Individuals referred to Post-COVID-19 outpatient clinic for medical assessment regardless of persistent symptoms or severity of COVID-19.	- Aged > 18 years with and without ongoing symptoms who recovered from COVID-19; - Confirmed by a PCR test within the last 6 months.	Pre-existing concomitant lung disease such as chronic obstructive pulmonary disease, asthma or interstitial lung disease.	Clinic patients

**Durstenfeld 2022 *(A)*[49]**	*Role of antibodies, inflammatory markers, and echocardiographic findings in post-acute cardiopulmonary symptoms after SARS-CoV-2 infection*	United States	The objective of this study was to determine whether echocardiographic findings, cardiac biomarkers, and inflammatory biomarkers obtained months after acute COVID-19 are associated with persistent cardiopulmonary symptoms.	Cohort study with control group	November 2020	May 2021	UCSF Division of Cardiology, NIH, National Heart Lung Blood Institute and National Institute of Allergy and Infectious Diseases, National Center for Advancing Translational Sciences	ORIGINAL STUDY (LIINC): Adults with documented history of SARS-CoV-2 RNA positivity at 2 weeks past onset of Covid-19 symptoms or, if asymptomatic, first positive test.<br>THIS STUDY: Adults >8 weeks after PCR-confirmed SARS-CoV-2 infection.	Used convenience sampling. Not clear.	- Pregnancy;<br>- Prior cardiovascular disease.	Voluntary
**Peluso 2022 *(A)*[62]**	*Plasma markers of neurologic injury and systemic inflammation in individuals with self-reported neurologic post-acute sequelae of SARS-CoV-2 infection (PASC)*	United States	To determine the relationship between LC symptoms and markers of neurologic injury.	Cohort study with control group	April 2020	NA	NIH	Volunteers (LIINC study).	At least one symptom of neurologic LC at 90 days post infection.	Lack of paired early (54 days) and late (121 days) samples.	Voluntary
**Singh 2021[39]**	*Persistent Exertional Intolerance After COVID-19: Insights From Invasive Cardiopulmonary Exercise Testing.*	United States	Explore the pathophysiologic mechanism of exercise intolerance for LC patients without cardiopulmonary disease or anaemia using invasive cardiopulmonary exercise testing (iCPET).	Case control study	February 2021	June 2021	None	1) Post C19 patients (10): Mean age 48 years ¬±15, 9 female, 1 male, average interval between positive PCR to iCPET was 11 months;<br>2) Controls age sex matched, unclear how (10): Mean age 48 years ¬±8, 8 female, 2 male.	- Post C19 patients - negative PCR at the time of the iCPET.<br>- Normal resting right heart hemodynamic values.	Two patients excluded during enrolment: one with long-standing history of fibrotic interstitial lung disease and one who exhibited iatrogenic chronotropic incompetence from B-adrenergic blocker therapy.	Clinic patients
**Matheson 2022[66]**	*Persistent 129Xe MRI Pulmonary and CT Vascular Abnormalities in Symptomatic Individuals with Post-Acute COVID-19 Syndrome*	Canada	To determine the relationship of persistent symptoms and exercise limitation with 129Xe MRI and CT pulmonary vascular measurements in individuals with LC.	Cohort study with control group	April 2021	October 2021	None	1) Study participants with a proven positive PCR COVID-19 test (prospectively recruited from a quaternary-care COVID-19 clinic); 2) Controls recruited via a convenience sample.	- Age ≥ 18 and < 80 year.s<br>- Documenyed case by positive RT-PCR test of COVID-19. resulting in post-infection symptoms.	- Contraindications to MRI such as implants and severe claustrophobia;<br>- Mental incapacitation, inability to give consent;<br>- Pregnancy.	Clinic patients
**Littlefield 2022[59]**	*SARS-CoV-2-specific T cells associate with reduced lung function and inflammation in pulmonary post-acute sequalae of SARS-CoV-2*	United States	To determine the frequency and function of SARS-CoV-2 specific T cells in blood and their relationship with plasma inflammatory markers and lung function in patients with Pulmonary LC.	Case control study	July 2020	April 2021	None	Unclear, but probably a combined in patient and outpatient LC population with an 'age matched' control group without LC.	- Positive PCR test;<br>- More than 6 months of defined LC pulmonary symptoms, cough, breathlessness, fatigue, exercise intolerance and hypoxia.	- Chronic or active infection;<br>- anti-inflammatories or antibiotics in past month.	Clinic patients
**Maamar 2022[60]**	*Post-COVID-19 syndrome, low-grade inflammation and inflammatory markers: a cross-sectional study*	Spain	To assess whether LC patients present higher levels of inflammatory markers after mild COVID-19; assess the hypothesis that chronic, low-grade inflammation (LGI) is a pathophysiological mechanism of LC. Secondary aim: assess gender differences in variables related to LC development.	Prospective cohort study	After September 2020	Not stated	None	Individuals with mild COVID-19 (none hospitalised), 3 months after the acute phase (median 115 days); none vaccinated.<br>PCS subgroup identified using NICE definition of LC;<br>Other participants constituted the control group.	- Confirmed COVID-19 cases by a positive result RT-PCR or by the presence of anti-SARS-CoV-2 IgG, three months after the acute COVID-19;<br>- A mild course of infection, according to the WHO definition, and characterized by fever, malaise, cough, upper respiratory symptoms, and/or less common features of COVID-19, in the absence of dyspnea.	None	Prospective recruitment from a single family medicine centre
**Beaudry 2022[45]**	*Persistent dyspnea after COVID-19 is not related to*	Canada	To examine whether cardiopulmonary mechanisms	Case control study	March 2021	August 2021	None	1) 28 people with self-reported ongoing symptoms >12 weeks;	12+ weeks from first molecular test positivity	- Diagnosis of PH predating COVID-19;	Clinic patients

	cardiopulmonary impairment; a cross-sectional study of persistently dyspneic COVID-19, non-dyspneic COVID-19 and controls		could explain exertional dyspnea in Long-CoV.					2) 24 people with no history of COVID-19;<br>3) 14 people declared recovered from COVID-19.	(mean time from test positivity to first research visit = 219 ¬± 82 days) and experiencing at least one persistent symptom at time of testing.	- Absolute contraindication to exercise testing or orthopedic limitation;<br>- Age <18 or >65 years;<br>- Body mass index > 30 kg/m2.	
**Giron 2022[57]**	*Markers of fungal translocation are elevated during post-acute sequelae of SARS-CoV-2 and induce NF-kappaB signaling*	Not stated	To examine the translocation of microbes — bacteria or fungus — is related to inflammation during PASC.	Other: cohort stdy with control group	Not stated	Not stated	Campbell Foundation grant, Commonwealth of Pennsylvania - COVID-19 funding; NIH grants; LIINC study supported by NIH/National Institute of Allergy and Infectious Diseases; Abbott Laboratories grant.	1) Subset of participants from LIINC study and Rush long Covid cohort;<br>2) 50 control of unknown origin.	Symptoms >12 weeks.	Not stated	- LIINC study<br>Rush long Covid cohort<br>- Controls of unknown origin
**Martini 2022[74]**	*Time-dependent recovery of brain hypometabolism in neuro-COVID-19 patients*	Italy	To determine the persistence of functional imaging abnormalities in neurocognitive LC.	Cohort study with control group	October 2020	November 2021	NA	1) Clinic patients (26);<br>2) Healthy controls (125) from a population cohort.	- New onset of neurological symptoms;<br>- Positive RT-PCR.	No stated	1) Cross-sectional approach<br>2) Prospectively recruited in the Neurology Unit of Ospedale Santo Stefano Prato, Italy<br>3) 125 healthy controls (HC) included from the AIMN (Associazione Italiana di Medicina Nucleare ed Imaging Molecolare) database on the AIMN websit
**Flaskamp 2022[87]**	*Serum of Post-COVID-19 Syndrome Patients with or without ME/CFS Differentially Affects Endothelial Cell Function In Vitro*	Germany	To determine alterations in EC function in vitro following their exposure to post-COVID-19 syndrome (PCS) patient serum including individuals with or without ME/CFS, i.e., PCS/CFS and PCS,	Case control study	November 2020	February 2021	Charité Chronic Fatigue Center (CFC) of the Charité Universitätsmedizin Berlin. L.F. received a scholar-ship from the Lost Voices Foundation e.V	1) Long Covid (PCS) patients (with and without CFS): mild to moderate disease course, persistent fatigue and exertion intolerance for at least 6 months, recruited within an ongoing observational study (PCS group: Mean age 42 94% female, Mean 8.3 months since infection<br>PCS/CFS group: Mean age 43, 85% female, Mean 9.4 months since infection);<br>2) Healthy controls: age- and sex-matched subjects.	LC patients:<br>- Persistent fatigue and exertion intolerance 6+ months after infection;<br>- previous SARS-CoV-2 infection confirmed by PCR or serology (IgG/IgA);<br>- Subgroup with ME/CFS met criteria for fatigue, post-exertional malaise (PEM), sleep dysfunction and pain, suffered from at least two neurological/cognitive manifestations, and suffered from at least one symptom of two categories - autonomic, neuroendocrine, and immune manifestations.	Pre-existing comorbidities, including fatigue.	Population survey
**Izzo 2022[41]**	*Combining L-Arginine with vitamin C improves long-COVID symptoms: The LINCOLN Survey*	Italy	To test the hypothesis that supplementation combining L-Arginine (to improve endothelial function) and Vitamin C (to reduce oxidation) could have favourable effects on LC symptoms.	Cross-sectional survey with two treatment arms (not described as randomised) after 30 days of treatment	No stated	Not stated	None	LC patients (symptoms present 4 weeks after infection) divided into two treatment arms in 2:1 ratio:<br>1) 2 vials/day of L-Arginine 1.66 g in with 500mg of liposomal Vitamin C (mean age 55.4, male 49.8%, hospitalised 7.9%, mean 33.8 days since negative test);<br>2) Multivitamin combination of Vitamin B1, Vitamin B2, Nicotinamide, Folic Acid, Pantothenic acid, Vitamin B6,	- 18 years or older;<br>- Men and non-pregnant women;<br>- Previous diagnosis of COVID-19 confirmed by RT-qPCR;<br>- Negative COVID-19 confirmed by RT-qPCR 4+ weeks after infection;<br>- Presence of COVID-19 sequelae 4 weeks after initial infection.	- History of intolerance to L-Arginine, Vitamin C, or components of the multivitamin combination;<br>- Pregnancy or breastfeeding;<br>- Cancer;<br>- Current treatment for chronic pulmonary disease;<br>- Use of immunosuppressive	Clinic patients

								Vitamin B12 (mean age 55.5, male 51.8%, hospitalised 9.2%, mean 31.5 days since negative test).		drugs or cytotoxic chemotherapy within previous 3 weeks;<br>- Enrolled in treatment study for COVID-19 in the 30 days before survey.	
**Besteher 2022[75]**	*Larger gray matter volumes in neuropsychiatric long-COVID syndrome*	Germany	To investigate brain structural changes in relation to course and neuropsychiatric symptom burden in long-COVID.	Case control study	April 2021	September 2021	Ongoing project (Post-COVID Brain) - part funded by IZKF Jena.	1) 30 long-COVID patients with neuropsychiatric symptoms (2-16 months since COVID-19 onset with mean time of 8.65 months, mean age 47.5 years, female 56.7%);<br>2) 20 healthy controls with no prior infection with COVID-19 (mean age 43.0 years, female 50.0%).	Long-COVID patients:<br>- Confirmation through real-time qPCR result at acute phase;<br>- Symptoms of fatigue and/or depressed mood and/or impairment of memory and concentration;<br>- IQ above 80.<br>Healthy controls:<br>- No history of positive SARS-CoV-2 test and no typical COVID-19 symptoms since December 2019;<br>- Negative test for IgG antibodies due to previous infection (antibodies against nucleocapsid antigen);<br>- IQ above 80.	- Exclusion criteria for an MRI scan;<br>- History of major neurological diseases and unmedicated internal medical conditions, especially chronic inflammatory conditions;<br>- History of or current substance abuse disorder.	Clinic patients
**Schultheiss 2021[48]**	*From online data collection to identification of disease mechanisms: The IL-1s, IL-6 and TNF-alpha cytokine triad is associated with post-acute sequelae of COVID-19 in a digital research cohort*	Germany	To explore a cytokine signature in LC.	Cohort study with control group	Not stated	October 2021	German Research Foundation; Medical Faculty of the Martin-Luther University Halle.	DigiHero health cohort.	In the cohort and completing the SARS-CoV2 module.	Lack of confirmed SARS-CoV2 infection.	Voluntary
**Yu 2022[82]**	*Lung Perfusion Disturbances Detected with MRI in Non-Hospitalized Post-COVID-19 Individuals with Dyspnea 3 - 13 Months after the Acute Disease*	Sweden	To detect pulmonary perfusion disturbances in non-hospitalized post-COVID condition with persistent dyspnea 4-13 months after the disease onset.	Case control study	October 2020	May 2021	Swedish Heart and Lung Foundation; MedTech Labs; and a private donation.	Mean time from symptom onset to the exam was 7.7¬±3.6 months.<br>Mean age in patients and controls (46.5 and 44.1).<br>21% and 16% were females in patients and controls.<br>Mean BMI 26 and 25.<br>mMRC scores 2 and 1 in patients and controls.<br>6MWT 583 and 677 in patients and controls.	- History of past COVID-19 infection, verified by real-time PCR;<br>- Persistent dyspnea at enrolment.	- History of smoking for more than five years;<br>- Cardiovascular or pulmonary conditions requiring medical follow-up or treatment;<br>- Contra-indication to MRI scanning.	Patient network in Sweden
**Finlay 2022[88]**	*Persistent post-COVID-19 smell loss is associated with inflammatory infiltration and altered olfactory epithelial gene expression*	United States	To explore the relationship between olfactory biopsies and loss of smell with COVID-19.	Case control study	Not stated	Not stated	NIH	ENT clinic patients.	Post LC anosmia/parosmia at 4 months.	Sinusitis of sinus disease.	Clinic patients
**Talla 2022[63]**	*Persistent serum protein signatures define an inflammatory subset of long COVID*	United States	To determine if serum proteome clusters predicted Long Covid.	Cohort study with control group	Not Stated	Not stated	None	Participants enrolled in the longitudinal study, "Seattle COVID-19 Cohort Study" to Evaluate Immune Responses in Persons at Risk and with SARS-CoV-2 Infection.	Adults in the greater Seattle area at risk for SARS-CoV2 infection or those diagnosed with SARS-CoV-2by a commercially available SARS CoV-2 PCR assay.	Not stated	Voluntary
**Patterson 2021[71]**	*Persistence of SARS CoV-2 S1 Protein in CD16+ Monocytes in Post-Acute Sequelae of COVID-19*	United States	To assess the kinetic differences in the proportions of monocyte subsets in severe COVID-19 cases, LC patients,	Case control study	Not stated	Not stated	Not clearly stated	Long COVID patients with symptoms 6 to 15 months after infection, severe COVID-19 cases and healthy controls.	- Post-acute COVID-19: symptoms extending beyond 3 weeks from initial onset;	Not stated	Not clearly stated, but most likely volunteers

	(PASC) up to 15 Months Post-Infection		and healthy controls; assess the presence of SARS-CoV-2 protein unaccompanied by corresponding viral RNA in CD14lo, CD16+ monocytes in LC patients up to 15 months after infection.					Study mentions also enrolling mild-moderate COVID-19 patients.	- Chronic COVID-19: symptoms extending beyond 12 weeks from initial onset.		
**Kravchenko 2021[81]**	*Cardiac mri in patients with prolonged cardiorespiratory symptoms after mild to moderate covid-19*	Germany	To explore the relationship between chronic COVID-19 syndrome (CCS) and myocardial injury and inflammation as an underlying cause of the persistent complaints in previously healthy individuals.	Case control study	January 2021	April 2021	German Heart Foundation and German Foundation of Heart Research; Grant from the BONFOR research program of the University of Bonn	1) 41 with Long Covid: mean age 39 years ±13, 18 men, 23 women. Median time between C19 and study MRI was 103 days, initial severity of C19 was mild (37, 90%) or moderate (4, 10%);<br>2) 42 controls: mean age 39 years ±16, 26 men, 13 women, no prior C19 infection.	- Over 18 years old;<br>- Recovery after C19 confirmed by PCR test;<br>- Resolution of acute C19 symptoms;<br>- Cardiorespiratory symptoms typical of Long Covid;<br>- Normal echocardiogram and electrocardiography;<br>- Negative trop.-T.	- History of cardiac disease;<br>- Chronic medical conditions e.g. hemochrombtosis, Fairy's disease, sarcoidosis, lupus, dermatomyositis, type 1 diabetes, COPD, hyper/hypo-thyroidism, rheumatoid arthritis;<br>- Contrast medium allergy;<br>- Metal intrauterine devices;<br>- Pregnancy/ breast feeding;<br>- Osteosynthesis material 25+cm in length;<br>- Cardiac pacemakers.	Participants referred by local medical offices and university centres
**Lee 2022[53]**	*EWAS of post-COVID-19 patients shows methylation differences in the immune-response associated gene, IFI44L, three months after COVID-19 infection*	Norway	(EWAS = epigenome wide association study)<br>Primary objective to compare the DNAm profles of the study participants.	Cohort study with control group	2021	2021	Research Council of Norway Grant	Sample drawn from 116,678 participants taking part in the Norwegian Corona Cohort Study<br>1) COVID-19 positive (n=109)<br>2) COVID-19 negative (n=73)<br>3) severe COVID-19 (n=61)<br>4) mild COVID-19 (n=48)<br>5) long-COVID (n=41)<br>6) remission (n=63)	- Lived in greater Oslo area;<br>- Categorised into 3 groups:<br>***Severe COVID-19 Participants*** with a positive SARS-CoV-2 RT-PCR test who had either been hospitalized because of COVID-19 or reported specific symptoms;<br>***Mild COVID-19 Participants*** with a positive SARS-CoV-2 RT-PCR test not requiring hospitalization and who had specific symptoms;<br>***Controls Tis group*** consisted of both symptomatic and healthy controls. Those who had a negative SARS-CoV-2 RT-PCR test and who reported all of the specific symptoms at the time of testing:. The healthy control group reported none of the specific symptoms during the three weeks preceding inclusion into the study:, and no infections during the past six months.	Not stated	Voluntary
**Galan 2022[89]**	*Persistent Overactive Cytotoxic Immune Response in a Spanish Cohort of Individuals With Long-*	Spain	To analyse the usefulness of several demographic, clinical, and immunological parameters (indicating inefficient/impaired cytotoxic	Case control study	Unclear: participants infected in March-April 2020 during	Not stated	National Center of Microbiology; Chiesi Espana, S.A.U.; Spanish	50 individuals who experienced mild symptomatic COVID-19 during first pandemic wave in Madrid:<br>1) LC patients (n=30): with signs	For all:<br>- Over 18 years old;<br>- Positive RT-qPCR assay for SARS-CoV-2 in nasopharyngeal smear or	None	Blood samples collected in the Primary Healthcare Center. Individuals with Long COVID recruited in collaboration with

	COVID: Identification of Diagnostic Biomarkers		response) as diagnostic biomarkers of Long-COVID.		first wave in Madrid		AIDS Research Network; NIH; Instituto de Salud Carlos III; Spanish Ministry of Science and Innovation	and symptoms of Long COVID 49 weeks post-infection (one individual showed an absence of detection of RNA from SAR-CoV-2 in blood);<br>2) Controls (n=20): recovered completely from COVID-19 within the first 12 weeks.	positive titers of virus-specific IgG;<br>- Did not require hospitalisation while with COVID-19;<br>- Infection prior to vaccination.<br>Long COVID patients:<br>- Experienced mild COVID-19;<br>- Have 8+ clinical signs and symptoms of LC according to NICE guideline at least 4 weeks after diagnosis.		Spanish Association of Patients with Long-COVID
**Sollini 2021 (*A*)[40]**	*Vasculitis changes in COVID-19 survivors with persistent symptoms: an [18F]FDG-PET/CT study*	Italy	To evaluate if 2-deoxy-2-[18F]fluoro-D-glucose ([18F]FDG) was able to demonstrate a persistent inflammatory process in the vascular epithelium or in any other site.	Non-comparative study for exploratory research	June 2020	Not stated	Not stated	Recovered adult COVID-19 patients, who complained of unexplained persisting symptoms for 30+ days during follow-up visits (regardless of severity of acute infection).	Not stated	None	Clinic patients
**Klein 2022[64]**	*Distinguishing features of Long COVID identified through immune profiling*	United States	To interrogate the biological underpinnings of Long COVID	Systematic review	Not stated	Not stated	Not clear	Groups recruited in separate clinical settings, prospectively.	Long COVID group (LC):<br>- Age ,> 18 years;<br>- Previous confirmed or probable COVID-19 infection;<br>- persistent symptoms > 6 weeks following initial COVID-19 infection.<br>Healthy Controls (HC):<br>- age ,>18 years;<br>- no prior COVID-19 infection;<br>- completion of verbal; screening with research staff confirming no active symptomatology.<br>Convalescent Group (CC):<br>- age ,> 18 years;<br>- Previous confirmed or probable prior COVID-19 infection;<br>- Completion of verbal screening with research staff confirming no active symptomatology.	- Inability to provide informed consent;<br>- any condition preventing a blood test from being performed.	Clinic patients
**Fancourt 2022[43]**	*Psychological consequences of long COVID: comparing trajectories of depressive and anxiety symptoms before and after contracting SARS-CoV-2 between matched long- and short-COVID groups*	UK	To explore mental health trajectories comparing LC v controls.	Cohort study with control group	March 2020	November 2021	Nuffield Foundation	Adults from the UCL Covid-19 Social Study living in England who reported contracting SARS-CoV-2 by November 2021 (N=3,115).<br>Of these, 15.9% reported having had long Covid (N=495).<br>Matched to participants who had short Covid using propensity score matching on a variety of demographic, socioeconomic and health covariates (N=962, n=13,325).	All participants living in England who completed the special module on Covid-19 experience in November 2021.	- Missing data on Covid-19 specific measures (1%);<br>- Reported infection with SARS-CoV-2 before 21st March 2020;<br>- Reported having Covid-19 more than once.	Voluntary
**Grist 2022[56]**	*The Investigation of Pulmonary Abnormalities using Hyperpolarised Xenon Magnetic Resonance*	UK	Establish whether lung abnormalities (not apparent on CT) that could cause/contribute to	Case control study	N/K	N/K	Oxford NIHR BRC; National Consortium of Intelligent	Long Covid patients (1 & 2 below) recruited from a Post-COVID Assessment clinic:<br>1) Post hospitalised patients: 2	Post hospitalised COVID patients:<br>- PCR proof of SARS-CoV-2 infection.	Post hospitalized:<br>- Not intubated;<br>- 3+ months post discharge;	Clinic patients

	Imaging in Patients with Long-COVID		breathlessness and are present in post-hospitalised COVID-19 patients are also present in non-hospitalised patients with Long Covid.				Medical Imaging; NIHR British Heart Foundation Oxford Centre of Research Excellence	female, 10 male, aged 57 ± 12 years, mean time from infection = 149 ± 68 days;<br>2) Non-hospitalised patients: 7 female, 4 male, aged 43 ± 11 years, mean time from infection = 287 ± 79 days;<br>3) Healthy: 6 female, 7 male, aged 41 ± 11 years.	Non-hospitalised LC patients:<br>- PCR or positive antibody proof of SARS-CoV-2 infection;<br>- LC diagnosed after referral to a specialist clinic with medically unexplained dyspnoea.	- No prior history of interstitial lung or airway disease (except mild well-controlled asthma with no evidence of airways obstruction);<br>- No smoking history of 10+ pack years;<br>- Normal/near-normal CT.<br>Non-hospitalized:<br>- No evidence of interstitial lung or airway disease (except mild well-controlled asthma with no evidence of airways obstruction);<br>- No smoking history of 10+ pack years;<br>- Normal/near-normal CT.<br>Healthy controls:<br>- No previous evidence of COVID-19 infections with PCR testing;<br>- No significant history of lung or cardiovascular disease or smoking.	
**Dennis 2022[44]**	*Multi-organ impairment and Long COVID: a 1-year prospective, longitudinal cohort study*	UK	Assess:<br>1) Symptoms, organ impairment and function over 1 year, particularly relating to ongoing breathlessness, cognitive dysfunction and HRQoL;<br>2) Associations between symptoms and organ impairment.	Cohort study	497 patients: Jan. 2020<br>39 patients: Sep 2020	497 patients: Sep 2020<br>39 patients: end date not defined	Horizon 2020 grant; AB: NIHR (including STIMULATE-ICP study), AstraZeneca, European Union, UKRI and BMA<br>DW: NIHR<br>MG: ARC, DH&SC, NIHR	Mean age 45 years, 73% female, median BMI 25 kg/m2, 13% COVID-19 hospitalisation, 32% healthcare workers.	- Prior SARS-CoV-2 infection;<br>- written informed consent.	- Active respiratory infection symptoms;<br>- Hospital discharge in last 7 days;<br>- Asymptomatic or hospital discharge > 4 months prior to enrolment;<br>- contraindications to MRI.	Clinic patients
**Roca-Fernandez 2022[42]**	*Cardiac impairment in Long Covid 1-year post-SARS-CoV-2 infection*	UK	To determine the trajectory of cardiac impairment (as measured by cardiac MRI) in subjects with LC.	Cohort study with control group	May 2020	August 2021	Amendment to a European Commission's Horizon 2020 grant	Volunteer population of those self-identifying as having Long Covid.	All	- Symptoms of active respiratory viral infection;<br>- Hospital discharge in the last 7 days;<br>- Contraindications to MRI.	Voluntary
**Peluso 2022 *(B)*[90]**	*Chronic viral coinfections differentially affect the likelihood of developing long COVID*	United States	To determine the relationship between Epstein-Barr virus reactivation, Cytomegalovirus and Human Immunodeficiency virus and LC symptoms.	Cohort study with control group	Not stated	Not stated	National Institute of Allergy and Infectious Diseases	Volunteers who tested positive at 2 academic medical centres, including clinicians and self referrals.	- Adult with a history of SARS-CoV-2 infection identified on nucleic acid amplification testing;<br>- Regardless of the presence of acute or postacute symptoms, was more than 14 days following symptom onset and followed approximately every 4 months thereaft.	Not stated	Voluntary

**Durstenfeld 2022 (*B*)[50]**	*Reduced Exercise Capacity, Chronotropic Incompetence, and Early Systemic Inflammation in Cardiopulmonary Phenotype Long Coronavirus Disease 2019*	United States	To compare symptomatic and recovered individuals using non-invasive cardiopulmonary exercise testing.	Cohort study	Not stated	Not stated	Philanthropic, NIH and intramural	46 individuals testing positive for COVID-19, 25 with symptoms, 21 recovered, all participants had new symptoms without alternative cardiopulmonary explanations and were more than 3 months after SARS-CoV-2 infection.	- PCR positive;<br>- Echocardiogram and CPET.	- Pregnancy;<br>- History of prior cardiopulmonary disease.	Population survey
**Maes 2022[55]**	*Lowered Quality of Life in Long COVID Is Predicted by Affective Symptoms, Chronic Fatigue Syndrome, Inflammation and Neuroimmunotoxic Pathways*	Thailand, Bulgaria, Iraq, Poland	To determine if affective symptoms in LC were mediated by inflammation in the acute phase and after.	Cohort study with control group	September 2021	December 2021	None	Acute COVID population from a variety of hospitals, both in and out patients.	- Daily life activities influenced by at least two symptoms;<br>- Symptoms lasting for 2+ months;<br>- Symptoms persisting beyond acute phase or become apparent 2-3 months later.	- Prior psychiatric diagnosis;<br>- Prior long term condition with inflammation or neurodegeneration.	Clinic patients
**Visvabharathy 2021[76]**	*Neuro-PASC is characterized by enhanced CD4+ and diminished CD8+ T cell responses to SARS-CoV-2 Nucleocapsid protein*	United States	To determine the contribution of virus-specific T cell responses to the etiology of chronic Neuro-Long Covid.	Case control study	February 2020	Not stated	Intramural	Participants prior to SARS-CoV-2 vaccination drawn from the 79 Neuro-COVID-19 outpatient clinic at Northwestern Memorial Hospital or from the surrounding 80 Chicago area.	1) Neuro-PASC patients meeting Infectious Disease Society of America clinical 83 criteria for COVID-19;<br>2) Neurological symptoms lasting at least 6 weeks.	Not stated	Neuro-COVID-19 outpatient clinic at Northwestern Memorial Hospital or from the surrounding 81 Chicago area
**Munker 2022[54]**	*Pulmonary function impairment of asymptomatic and persistently symptomatic patients 4¬†months after COVID-19 according to disease severity.*	Germany	To explore pulmonary function impairment after COVID-19.	Cohort study with control group	March 2020	August 2020	None	Patients recruited from a hospital outpatient department.	- Age > 18 years;<br>- LMU outpatients department for 4 month follow up;<br>- Confirmed positive RT-PCR or positive Anti-SARS-CoV-2 Immunoglobulin G (IgG) titer.	Not stated	Clinic patients
**Peluso 2021 (*C*)[61]**	*Markers of Immune Activation and Inflammation in Individuals With Postacute Sequelae of Severe Acute Respiratory Syndrome Coronavirus 2 Infection*	United States	To measure soluble markers of systemic immune activation and inflammation in a SARS-CoV-2 recovery cohort at early (<90 days) and late (>90 days) timepoints and to track the longitudinal changes in these markers for those with and without LC at the late timepoint.	Cohort study	Not stated	Not stated	NIAID; NIH; Zuckerberg San Francisco General Hospital Department of Medicine and Division of HIV, Infectious Diseases, and Global Medicine; UCSF Resource Allocation Program.	Volunteers with a documented history of SARS-CoV-2 infection confirmed by nucleic acid amplification testing. 27 participants (22.3%) had been hospitalised; of these, 9 were managed in ICU and 3 required mechanical ventilation.<br>None were vaccinated prior to the data collection for the study.	All adults with a positive test were eligible and recruitment was agnostic to the presence of persistent symptoms.	HIV patients.	A combination of clinician referrals, mailings to consecutive patients testing positive at university-affiliated testing sites, and responses to medical center recruitment postings and websites
**Sollini 2021 (*B*)[47]**	*Long COVID hallmarks on [18F]FDG-PET/CT: a case-control study.*	Italy	The present study hypothesised that whole-body [18F]FDG-PET/CT might provide insight into the pathophysiology of long COVID.	Case control study	Not stated	Not stated	Italian association for Cancer Research	1) Patients in an outpatient clinic;<br>2) Controls: pre-existing scans in melanoma patients.	At least one persistent symptom more than 30 days post recovery.	None	Clinic patients
**Guo 2022[58]**	*COVCOG 2: Cognitive and Memory Deficits in Long COVID: A Second Publication From the COVID and Cognition Study.*	UK	To objectively assess cognitive performance in those with LC and self reported cognitive dysfunction. Researchers also hypothesised that some symptom profiles may be more predictive of cognitive performance thanothers, perhaps giving some information about the mechanism.	Case control study	Not stated	Not stated	This study was not supported by any funding bodies but did benefit from research funds from the Department of Psychology, University of Cambridge	Aged 18 and over	Experienced COVID-19 or may have experienced COVID 19 (not confirmed.	Not stated	Recruited through word of mouth, student societies, and online/social media platforms such as the Facebook Long COVID Support Group and the Prolific recruitment site.

**Chudzik 2022[83]**	*Chronic Fatigue Associated with Post-COVID Syndrome versus Transient Fatigue Caused by High-Intensity Exercise: Are They Comparable in Terms of Vascular Effects?*	Poland	To see if chronic fatigue associated with post-COVID is associated with permanent vascular dysfunction caused by initial vascular injuries.	Case control study	Not stated	Not stated	European Regional Development Fund under the Smart Growth Operational Program	1) Patients with ongoing symptoms lasting at least 4 weeks after last symptom of infection;<br>2) Matched healthy volunteers with no covid infection history;<br>3) Non-matched highly trained amateur runners.	Group 1: Covid-19 with ongoing symptoms for at least four weeks;<br>Group 2: No past history of covid-19;<br>Group 3: Free of illness/injury.	Group 3: Medication.	Voluntary
**Gorecka 2022[46]**	*Cardiovascular magnetic resonance imaging and spectroscopy in clinical long-COVID-19 syndrome: a prospective case,Äìcontrol study*	UK	Assess cardiac involvement in long COVID-19 syndrome in previously healthy and non-hospitalised patients. Combining CMR and 31P-CMRS in an observational prospective case–control study.	Case control study	March 2021	July 2021	Welcome Trust Clinical Career Development Fellowship	Long Covid participants:<br>1) Mean age of 45 years ± 13, 9 male, 10 female, mean duration of symptoms at time of assessment = 163 days.<br>Controls:<br>2) Mean age of 51 years ± 11, 6 male, 4 female, no previous Covid 19 diagnosis.<br>(Note; PCR testing was not done to exclude asymptomatic Covid-19 infection nor antibody testing to potentially exclude prior infection.)	- Long COVID symptoms;<br>- Continued symptoms for more than 12 weeks;<br>- Alternative diagnosis have been excluded;<br>- Seropositive.	- Patients with known coronary artery disease, cerebrovascular disease, cardiac surgery, atrial fibrillation, moderate or above valvular heart disease, hypertension, any type of diabetes, renal impairment, COPD;<br>- Resolution of symptoms at the time of assessment;<br>- Contraindications to CMR.	Clinic patients
**Clark 2021[80]**	*Cardiovascular magnetic resonance evaluation of soldiers after recovery from symptomatic SARS-CoV-2 infection: a case,Äìcontrol study of cardiovascular post-acute sequelae of SARS-CoV-2 infection (CV PASC)*	United States	To evaluate the spectrum of cardiac involvement among soldiers with cardiopulmonary symptoms in the late convalescent phase of recovery from SARS-CoV-2 compared to a healthy soldier control group, and to determine the rate of progression to cardiovascular sequelae of SARS-CoV-2 (CV PASC).	Cohort study	March 2020 (assumed)	Not stated	National Heart, Lung and Blood Institute of the NIH	Serving soldiers.	Controls:<br>- Normal cardiac function without myocardial pathology.<br>Cases and controls:<br>- Active duty or recent military retirement in the prior year.	No stated	Population survey
**Crunfli 2022[73]**	*Morphological, cellular, and molecular basis of brain infection in COVID-19 patients*	United States	To gain further insight into the neuropathological and neurological consequences of COVID-19 and possible cellular and molecular mechanisms.	Cohort study with control group	Not stated	Not stated	Not stated	1) 81 patients 57 (SD26) days after SARS-CoV-2 detection;<br>2) 81 healthy volunteers;<br>3) 12 brain samples (SARS-CoV-2 positive);<br>4) 8 brain samples (SARS-CoV-2 negative).	Not specified	Not specified	Postmortem brain samples

Individual Papers – Review 2, PART 2												
**Study ID**	**Total no. of Participants**	**Long Covid: Hospitalised**	**Long Covid: Not Hospitalised**	**Long Covid Overall**	**Controls: Hospitalised**	**Controls: Not hospitalised**	**Controls: Overall**	**Symptom Data Gathered**	**Pathophysiological Data Gathered**	**Summary of Principal Positive Findings**	**Summary of Principal Negative Findings**	**Relationship Supported**
**Holmes 2021[52]**	86:<br>1) Healthy control group (n = 41);<br>2) hospitalized patient group (n = 18);<br>3) Recovery cohort consisting of a non hospitalized group sampled 3 months post the acute phase of the disease (n = 27) with acute-phase symptoms and symptoms 6 months post COVID infection.	Unclear	Unclear	Unclear	Unclear	Unclear	Unclear	In addition to persistent respiratory symptoms including cough, dyspnea, chest pain and rhinorrhea, there were also non-respiratory "systemic" symptoms including fatigue, anosmia, myalgia, abdominal pain, and joint pain	- Lipoprotein reports itemizing 112 lipoprotein parameters for each plasma sample generated using the Bruker IVDr Lipoprotein Subclass Analysis<br>- α1-acid glycoprotein N-acetyl-glucosamino (N-acetyl) signal integrals were calculated<br>- Fully quantitative amino acid analysis of 35 molecular species<br>- Quantitative analysis of tryptophan and 14 catabolites in its metabolic pathway (15 pathways) was performed	• Clear metabolic patterns associated with persistent inflammatory and metabolic derangements in a majority of non hospitalized 3-month-post-COVID-19 patients, with one or more symptoms being persistent in 57% up to 6 months following the acute phase<br>• Lipoproteins' parameter variance was significantly greater for symptomatic than asymptomatic patients<br>• Increased levels of 3-indole acetic acid implies a microbiome functionality shift in the post- acute COVID-19 patients; may also be pertinent to understanding the tryptophan pathway perturbations in PACS patients given the general importance of the microbiome in tryptophan metabolism and serotonin production	Most metabolic parameters showed a high level of normalization	**Immunological disturbance (including inflammation)**
**Weinstock 2021[84]**	n=352:<br>1) Long Covid patients (n=136);<br>2) General population controls (n=136);<br>3) Mast cell activation syndrome (MCAS) patients (n=80)	Unclear	Unclear	136	Unclear	Unclear	136	- LC symptoms: presence and severity<br>- MCAS symptoms: presence and severity<br>- MCMRS questionnaire applied to all 3 groups<br>- Fatigue assessment scale (FAS)<br>- Self-rated general health/quality of life: range 0-100<br>- Medications and supplements taken<br>- Long Covid patients filled out questionnaires for pre-COVID status by recall and post-COVID status<br>- MCAS patients filled out questionnaires for pre-treatment status by recall	Comparison of MCA symptoms between Long Covid and MCAS patients to test the hypothesis of MCA as mediator of Long Covid symptoms	• Lack of significant difference in MCMRS score between controls and pre-COVID-19 status of LC patients<br>• Significant increase in MCMRS score between pre- and post-COVID-19 status for LC patients<br>• Lack of significant difference in MCMRS score between LC and MCAS patients<br>• Significantly higher number of body systems affected by MCA symptoms for Long Covid patients pre-COVID-19 status than controls. The number of affected systems significantly increased between pre- and post-COVID-19 and there was a lack of significant difference between LC and MCAS patients.<br>Similar results in comparisons of MCMRS symptom counts, MCMRS cumulative severity	See summary of principal positive findings for the mix of results for or against hypotheses	**Mast Cell Activation**

										scores and FAS scores. QoL scores did not significantly differ between controls and pre-COVID-19, increased significantly between pre- and post-COVID-19, but was significantly lower for LC patients vs. MCAS patients. Lack of significant difference in individual symptom severity scores for 48 of 51 symptoms between controls and pre-COVID-19 status of LC patients. Significant increases in all symptoms except seizures between pre- and post-COVID-19 status. Significant differences in severities of 21 of 51 symptoms between LC and MCAS patients.		
**Files 2021[38]**	50	10	10	20	0	30	30	Symptoms reported beyond 30 days in the prolonged group included dyspnea, fatigue, psychataxia, and/or cough. (Note: isolated anosmia/ageusia for more than 30 days did not meet criteria for classification into the prolonged group.) Antibody ELISA.	- Peripheral blood samples prior to any vaccination<br>- Immune cell subset frequencies by flow cytometry and phenotyping panels<br>- Expression of activation and exhaustion markers using incubation with S-protein peptides, flow cytometry and staining	• Individuals with prolonged symptom duration maintained antigen-specific T cell response magnitudes to SARS-CoV-2 spike protein in CD4+ and circulating T follicular helper cell populations during late convalescence, while those without persistent symptoms demonstrated an expected decline.<br>• The prolonged group also displayed increased IgG avidity to SARS-CoV-2 spike protein.<br>• Significant correlations between symptom duration and both SARS-CoV-2-specific T cells and antibodies were observed.	Activation and exhaustion markers in multiple immune cell types revealed few phenotypic differences between prolonged and recovered groups, suggesting that prolonged symptom duration is not due to persistent systemic inflammation, but possibly viral persistence	**Viral persistence**<br><br>**Immunological disturbance (including inflammation)**
**Glynne 2022[65]**	65	Not stated	Not stated	49	Not stated	Not stated	16	Symptom questionnaire was designed with a binary symptom grid to initially record the presence or absence of certain long-COVID symptom categories	Routine blood tests including FBC, ESR, CRP, D-dimer, renal and liver function tests.<br><br>Flow Cytometry performed on initial blood tests.<br><br>Antibody panel allowed comparison between expression levels (antigen densities) of proteins important for regulating T-Cell function.<br><br>Measured response to Histamine Receptor Antagonists (HRA) by comparing symptom profiles	• HRA treatment reduced average symptom burden by 59.7% . The mean time to response was 29.6 days (median 26 days; range 6 - 89 days). Patients reported improvements in all symptoms except dysautonomia<br>• Of the 17 long-COVID patients with a history of atopy, 11 received HRA, and of these 8 reported a clinical improvement<br>• 25/49 symptomatic and 3/16 asymptomatic participants had CD4+ EM counts that were below the lower limit of the normal range , and receiver operating	There was no correlation between SARS-CoV2 antibody status and response to HRA	**Immunological disturbance (including inflammation)**<br><br>**Mast Cell Activation**

									in treated and untreated cohorts.<br><br>The 24 patients who didn’t receive HRA were reassessed between 28 and 119 days after initial blood test for symptom changes.	characteristic (ROC) analysis confirmed that the CD4+ EM count could distinguish the two groups .<br>• 43/49 long-COVID patients and 14/16 of the asymptomatic group had reduced CD8+ EM counts, which were below the median of the normal range<br>• PD-1 (Programmed cell death protein 1, CD279) was significantly increased in both CD4+ and CD8+ CM cells in all participants, although this was more marked in symptomatic long-COVID patients		
**Apple 2022[37]**	32	0	22	22	0	10	10	- Neurocognitive interviews: presence of 23 different cognitive symptoms; cognitive PASC diagnosis if one or more new, persistent cognitive symptom(s) after acute illness with COVID-19<br>- Neuropsychological testing: applied the testing criteria for HIV-associated neurocognitive disorder (HAND)<br>- Time to cognitive symptom onset	- Pre-existing cognitive risk factors: hypertension; diabetes; sleep apnea; HIV; depression; anxiety; ADHD; learning disability; daily psychoactive medication; history of mild TBI; history of hypothyroidism; history of vitamin B12 deficiency; history of recurrent stimulant use; history of heavy alcohol use<br>- Lumbar puncture CSF collection: CSF-serum albumin ratio, a measure of blood brain barrier (BBB) permeability	• Participants with delayed onset (>1 month) of cognitive PASC were younger than those with acute onset of cognitive PASC (median age of 39 vs. 50 years<br>• Cognitive post-acute sequelae of LC participants had a higher number of pre-existing cognitive risk factors with no differences in the presence of specific cognitive risk factors<br>• Cognitive LC participants had higher proportion with abnormal CSF findings versus controls<br>• Abnormal oligoclonal banding (OCB) patterns were identified in 69% (of cognitive PASC participants compared to 0% of controls<br>• Cognitive risk factors and immunologic mechanisms may contribute to PASC pathogenesi	Neuropsychological testing revealed that 59% with cognitive LC met equivalent HIV-associated neurocognitive disorder (HAND) criteria for objective cognitive impairment, compared to 70% of cognitive controls<br><br>All participants conducting CSF collection had normal values for CSF white blood cells, glucose, calculated CSF/serum albumin ratio, IgG index, CSF IgG level, and serum IgG level and did not differ between participant groups	**Immunological disturbance (including inflammation)**<br><br>**(Large proportion of participants reporting a delayed onset of cognitive PASC implies that events occurring after the acute period of SARS-CoV-2 infection may contribute to pathogenesis and respond to early intervention)**
**Fogarty 2021[51]**	67	37	13	50	0	17	17	Clinical assessment at time of outpatient review included:<br>- chest x-ray<br>- 6-min walk test (6MWT), measuring distance covered, lowest arterial oxygen saturation, and maximal exertion (using a Modified Borg Scale)<br>- Fatigue scores assessed using Chalder fatigue scale	- Plasma VWF:Ag, VWFpp, FVIII:C and sTM<br>- Thrombin generation<br>- Release of extracellular DNA was measured using fluorescent DNA-intercalating dye Sytox Green (Invitrogen)<br>- DNase activity assessed by an in vitro NET degradation assay<br>- Activation of the contact factor pathway	• LC patients had increased thrombin generation<br>• Plasma FVIII:C levels remained significantly increased compared with controls<br>• VWF:Ag levels were significantly increased in convalescent COVID-19 patients compared with controls	No significant differences in systemic FXIIa levels between a subset of convalescent patients (n=20) and controls (n=17). Relationships between clinical factors and clotting factor changes were not significant on regression	**Endothelial dysfunction**<br><br>**Thrombin generation**
**Ferrando 2022[68]**	60	7	25	32	1	28	28	- CDC COVID-19 symptom score<br>- Lawton-Brody IADL	Proinflammatory cytokine levels from serological samples: - CRP<br>- IL-6	• Statistically significant difference in the number of individuals with elevated IL-6 and CRP levels	No significant correlations between TNF-alpha and	**Immunological disturbance (including inflammation)**

								- Chalder Fatigue Scale<br>- Psychiatric measures: PHQ-9, Endicott QLESQ, PCL-5, GAD-7<br>- Neuropsychological battery: PAOF, RBANS, NP tests for attention, immediate memory, delayed memory, visuospatial, language, Trail Making Test, Verbal fluency, Stroop Color Word score	- TNF-alpha	between clinical group and non-clinical control group<br>• Correlations between proinflammatory cytokines and medical, psychiatric, and NP variables:<br>- Significant positive correlation between IL-6 and Chalder Fatigue Scal<br>- Significant negative correlations between IL-6 and Stroop Color Word Test t-score and Trail Making Test Part B t-<br>- Significant positive correlations between CRP and current CDC COVID illness and PHQ-9<br>- Significant negative correlation between CRP and Endicott QLESQ	psychiatric and NP variables	
**Lehmann 2022[79]**	135	Unclear	Unclear	96	Unclear (30 overall)	Unclear	35	- Persistent respiratory symptoms: dyspnoea on exertion/limited exercise capacity; cough; thoracic pain/tightness of chest<br>- Other persistent symptoms: headache, dizziness and fatigue; dysgeusia and dysosmia; palpitations; joint pain	- Pulmonary function test (PFT), including FVC, FEV1 and TLC. Pathological findings: FVC <80%; TLC <lower limit of normal<br>- Diffusion capacity measurement: TLCO SB ans TLCO/VA. Pathological findings: TLCO SB <80%; TLCL/VA <80%<br>- Blood gas analysis (BGA): including inflammatory markers, D-dimer. Pathological findings: Fibrinogen > 400 mg/dl; (IL-6) > 7 pg/ml; CRP > 0.5 mg/dl; D-dimer > 0.5 µg/ml<br>- CT scan for radiological abnormalities	• Respiratory symptom group had significantly lower FVC (%), TLC (L) and TLCO SB (%) relative to symptom-free group<br>• In multiple logistic regression including variables that were significant in univariate analyses, age (P=0.004), TLCO SB (%) (P=0.042), and days since acute COVID-19 (P=0.017) were significant covariates in predicting respiratory symptoms<br>• Impairment of diffusion capacity indicates an interstitial lung disease consistent with CT results, which revealed pulmonary interstitial changes in 35% of the 117 patients<br>• [R]espiratory viral infection induces distinct fibroblast activation that might be responsible for the pulmonary interstitial changes and thus the reduced TLCO SB	The percentage of subjects with abnormal results in PFT, TLCO SB, TLVO/VA, BGA, laboratory tests and MDCT did not differ significantly between both groups<br><br>Associations with TLC (L) and FVC (%) were statistically insignificant in multivariate logistic regression for having respiratory symptoms	**Reduced tissue oxygen extraction**
**Durstenfeld 2022 *(A)*[49]**	102	6	41	47	13	42	55	Individuals queried re. presence of 32 individual symptoms from the CDC list of COVID-19 symptoms and from the Patient Health Questionnaire Somatic Symptom Scale.	- Echocardiograms<br>- Troponin I<br>- CRP<br>- NT-Pro-BNP<br>- IL-6<br>- IL-10<br>- IFN-gamma<br>- TNF-alpha<br>- RBD IgG	• Minor echocardiographic findings - 4/47 pericardial effusion<br>• 1 with myocarditis<br>• Raised CRP more frequent in LC group<br>• RBD Ig- G levels higher in LC	Echocardiography and cardiac MRI essentially normal<br><br>Troponin and TNF-alpha no difference	**Viral persistence**<br><br>**Immunological disturbance (including inflammation)**
**Peluso 2022 *(A)*[62]**	121	13	39	52	14	55	69	Memory/concentration issues,headache, vision	- Markers of neurologic injury (glialfibrillary acidic	Those who went on to report CNS LC had:	No differences in levels of markers at either recovery time point	**Immunological disturbance (including inflammation)**

								problems, dizziness, and balance issues.	protein [GFAP], neurofilament light chain [NfL])<br>- Soluble markers of inflammation<br>- Cytokine 3-PlexA, IL-6, IL-10, TNf-α, IFN-y, IP-10,<br>- monocyte chemo attractant protein 1 [MCP-1])<br>- SARS-CoV-2 receptor-binding	• significantly higher levels of:<br>• GFAP (1.3-fold higher mean ratio)<br>• higher levels of:<br>• cytokines IL-6 (1.48-fold higher mean ratio)<br>• TNF-α (1.19-fold higher mean ratio)<br>• chemokine MCP-1 (1.19-fold higher mean ratio)	between those with and without prior CNS symptoms during acute infection	**Neuropathology**
**Singh 2021[39]**	20	1	9	10	0	10	10	Unexplained exercise intolerance after recovering from COVID-19 without cardiopulmonary disease or anaemia	- CT scan of chest<br>- Pulmonary function test<br>- Resting echocardiography<br>- Invasive cardiopulmonary exercise testing (iCPET) data	LC patients showed (vs. controls):<br>• greater peak exercise mixed venous oxygen saturation (mean 50% vs 22%)<br>• greater peak venous O2 content (mean 33 vs 22 mmHg)<br>• greater degree of ventilatory inefficiency, i.e. abnormal ventilatory efficiency (VE/VCO2) slope	No evidence was found of parenchymal lung disease on chest CT imaging, and all patients demonstrated left ventricle ejection fraction of >50% with no evidence of moderate or severe valvular heart disease, no evidence of right-to-left intracardiac shunt defect on resting right heart catheterization and echocardiography, and no evidence of acute coronary syndrome defined by ST-segment elevation myocardial infarction, non-ST-segment elevation myocardial infarction, unstable angina, or a combination there of during exercise testing	**Reduced tissue oxygen extraction**
**Matheson 2022[66]**	40	12	22	34	0	6	6	- 6 minute walk test<br>- St. George's Respiratory Questionnaire (SGRQ)<br>- Modified Medical Research Council (mMRC)<br>- COPD Assesment Test<br>- Post COVID-19 Functional status scale<br>- International Physical Activity Scale<br>- Modified Borg Dyspnoea scale<br>- Fatigue, respiratory and cardiac symptoms, headache, cognitive problems	Participants with PACS:<br>- spirometry<br>- diffusing capacity of the lung for carbon monoxide (DLCO)<br>- 129Xe MRI<br>- chest CT<br>Healthy controls:<br>- spirometry<br>- DLCO<br>- 129Xe MRI<br>- SpO2<br>- heart rate	• Xe MRI RBC:barrier ratio correlated with DLCO and FEV1<br>• 129Xe MRI RBC AUC correlated with CT BV5, IPAQ score, post-exertional SpO2 and post-6MWT Borg breathlessness (but not SGRQ score)<br>• BV5 correlated with post-exertional SpO2<br>• Microvascular remodelling, shunting, thrombuses, micro-embolisms, or some combination of these may play a role	In all patients with COVID-19, mean spirometry values were normal and mean DLCO was at the bottom of the normal range	**Microclots**<br><br>**Endothelial dysfunction**
**Littlefield 2022[59]**	40	8	12	20	0	20	20	Symptom: Fever/Chills, Cough/Shortness of breath, Sore throat/Runny nose, Cardiac, Neurological/Cognitive, Fatigue, Smell/Taste changes, Gastrointestinal, Muscle aches	- Frequency of SARS-CoV-2-specific T cells using intracellular cytokine (IFN-y,TNF-α and IL-2) staining after stimulation with peptide pools of SARS-CoV-2 spike(S), nucleocapsid(N) or membrane (M) surface-expressed proteins	• SARS-CoV-2 specific T cells, IL-6 and CRP raised and related to both symptoms and specific T cells<br>• Indications of recent activation persist over 6 months		**Viral persistence**<br><br>**Immunological disturbance (including inflammation)**

									- Markers of T-cell maturation (CD27 and CD45RA)<br>- Plasma IL-6 and CRP			
**Maamar 2022[60]**	121	0	36	36	0	85	85	Symptoms: most reported symptoms were fatigue (42.8%), anosmia (40%), ageusia (22.8%), dyspnea (17.1%), myalgia (11.4%), and palpitations (11.4%)	- CRP serum levels<br>- Ferritin<br>- Lactate dehydrogenase (LDH)<br>- Fibrinogen<br>- D-dimer<br>- Neutrophil and lymphocyte counts and ratio (NL ratio)<br>Composite index of five markers created:<br>- Abnormal neutrophil count or NL ratio<br>- CRP in LGI range or abnormal fibrinogen level<br>- Abnormal neutrophil count or fibrinogen level<br>- Abnormal NL ratio or fibrinogen level<br>- CRP in LGI range or abnormal neutrophil count	• Significantly higher neutrophil count for:<br>• female LC patients with anosmia than female non-LC controls<br>• female patients with ageusia than controls<br>• male patients with fatigue than controls<br>• Significant odds ratios of PCS prevalence for: C1, C3, and C4 indices for women; C2, C5, and CRP in LGI range for men	Lack of significantly higher neutrophil count for:<br>• male LC patients with anosmia than male non-LC controls<br>• male patients with ageusia than controls<br>• female patients with fatigue than controls.	**Immunological disturbance (including inflammation)**
**Beaudry 2022[45]**	66	4	24	28	3	35	38	Symptoms: duration; dyspnea; fatigue. fever; amnosia; loss of taste; muscle pain; headaches; cough; diarrhea	- mMRC Dyspnea scale<br>- pulmonary function test<br>- incremental cardiopulmonary exercise testing	None	• VO2 peak, pulmonary function and cardiac/pulmonary vascular parameters not impaired compared to normative values and cardiopulmonary responses to exercise were otherwise normal<br>• When stratified by clinical dyspnea severity there were no between-group differences in VO2 peak<br>• During submaximal exercise, dyspnea and ventilation were increased in the mMRC>1 group, despite normal operating lung volumes, arterial saturation, diffusing capacity and indicators of pulmonary vascular pressures	**Viral persistence;**<br>**Other: None supported**
**Giron 2022[57]**	217	36	131	167	Unclear	Unclear	50	- Symptoms<br>- QoL evaluation	- Plasma markers of tight junction	• Significant difference in beta-glucan between Rush	Weak correlations between QoL scores and markers	**OMight be looking at endothelial dysfunction but no evidence to**

									permeability/microbial translocation<br>- plasma markers of inflammation<br>- metabolites	cohort PASC patients and control group<br>• Suggestion that beta-glucan pathway activation can be inhibited with the Syk inhibitor piceatannol		**support strong/primary link between translocation of microbes, bacteria or fungus as a primary pathway for ongoing symptoms post SARS-CoV-2 infection**
**Martini 2022[74]**	151	unclear	unclear	26	unclear	unclear	125	- Global cognitive functioning tested throughout the Mini-Mental State Examination (MMSE) in 19 out of 26 patients<br>- More extended neuropsychological evaluation obtained in a subgroup of patients (11/26)	FDG-PET imaging	• Hypometabolism of front-insular cortex improving over 9 months after acute COVID that correlated with clinical neurocgnitive deficits<br>• Hypermetabolism in brainstem, cerebellum, amygdala, hippocampus and parahippocampus that persists		**Neuropathology**<br><br>**Direct Neuropsychiatric effect**
**Flaskamp 2022[87]**	44	Unclear	Unclear	30 (Without CFS: 17 With CFS: 13)	Unclear	Unclear	14	In vitro data on endothelial cell (EC) functioning: autoantibody binding level; EC surface activation markers (E-Selectin, VCAM-1, ICAM-1, PECAM-1); EC secretion profile, specifically of molecules implicated in vascular inflammation (sVCAM-1, sICAM-1, IGFBP-4, MRP8/14, MPO, NGAL, Cystatin C, MMP-9, MMP-2); angiogenesis potential assessed by number of junctions and total mesh area	- Immune mediators that contribute to endothelial damage: anti-endothelial cell autoantibodies (AECA) and cytokines<br>- Human EC exposed to patient and control serums containing these and other mediators	• PCS/CFS subgroup showed significantly elevated level of IgG autoantibody binding compared to the control group for both endothelial cell types: macrovascular HUVEC and microvascular HDBEC (P=0.022)<br>• Statistically significant reduction in HUVEC activation marker VCAM-1 for both PCS and PCS/CFS relative to controls, in ICAM-1 for PCS/CFS relative to controls and PCS, in E-Selectin for both PCS and PCS/CFS (relative to controls<br>• Statistically significant increase in HUVEC activation marker PECAM-1 for both PCS and PCS/CFS relative to controls.<br>• Statistically significant differences in HUVEC EC secretion profiles between one or more patient subgroups and controls for sVCAM-1, sICAM-1, IGFBP-4, MRP8/14, MPO, NGAL, cystatin C, and MMP-9<br>• Significant difference between patient subgroups for IGFBP-4, MRP8/14 and cystatin C<br>• Statistically significant increase in HUVEC number of junctions for PCS subgroup relative to controls	Statistically insignificant differences in serum cytokine levels between controls and both patient subgroups, except for IL-18 between controls and PCS and sVEGFR between controls and PCS and between controls and PCS/CFS<br><br>No significant difference between patient subgroups in any cytokine	**Immunological disturbance (including inflammation)**<br><br>**Endothelial dysfunction**

**Izzo 2022[41]**	1390	7.9%	800	869 (L-Arginine + Vit. C group)	9.2%	473	521 (Multivitamin group)	- Physician assessed symptoms: fatigue, dyspnea, chest tightness, dizziness, gastrointestinal disorders, headache, anosmia, difficulties in concentrating, sleep disturbances<br>- Borg scale for effort perception	- Endothelial function improvement<br>- Oxidative stress reduction	• Statistically significant reductions in all physician-assessed symptoms for L-Arginine+Vit C group relative to multivitamin group<br>• Statistically significant decrease in Borg effort perception score for L-Arginine+Vit C group relative to multivitamin group		**Immunological disturbance (including inflammation)**<br><br>**Endothelial dysfunction**<br><br>**Oxidative stress**
**Besteher 2022[75]**	50	2	28	30	0	20	20	- Montgomery-Asberg Depression Rating Scale (MADRS)<br>- State-Trait-Anxiety Inventory (STAI)<br>- Montreal Cognitive Assessment (MoCA)	- Gray matter volume scanned by 3T-MRI<br>- whole-brain comparison by voxel-based morphometry	• Significantly enlarged gray matter volume (GMV) in LC patients present in several clusters (spanning fronto-temporal areas, insula, hippocampus, amygdala, basal ganglia, and thalamus in both hemispheres) when compared to controls<br>• Stepwise linear regression among LC patients identified time since onset of COVID-19 as a significant regressor in four of the GMV clusters with an inverse relationship<br>• Age and gender were significant predictors in all GMV clusters, suggesting a linear relationship with GMV	Stepwise linear regression among LC patients: No significant associations between GMV clusters and MADRS, STAI and MoCA as measures of neuropsychiatric symptoms	**Neuropathology**<br><br>**Direct Neuropsychiatric effect**
**Schultheiss 2021[48]**	318 (Breakdown of participants within the paper does not add up correctly.)	8	162	175	0	36	36	Prior selected representative LC symptoms	- Cytokine plasma levels measured using LEGENDplex Human B Cell Panel 171 (13-plex) and Human Anti-Virus Response Panel (13-plex) (BioLegend)<br>- Autoantibody screens: Rheumatoid Factor 173 (detects IgG, IgA and IgM RFs), ANA (detects SS-A 60, SS-A 52, SS-B, RNP-70, 174 Sm, RNP/Sm, Scl-70, centromere B and Jo-1 IgGs) and Anti-Phospholipid IgG/IgM 175 (detects cardiolipin, phosphatidylserine, phosphatidylinositol, phosphoglycerides and 176 β2-glycoprotein 1 IgGs/IgMs) - Single Cell transcriptomic studies: Cytokine response scores.	• Cytokine profiling revealed a significant association of a well-known triad of cytokines - IL-1β, IL-6 and TNF-α with PASC<br>• Analysis of macrophages from the bronchoalveolar lavage fluid in patients with severe COVID-19 showed a specific pro-inflammatory macrophage subset produced high levels of these cytokines in acute disease<br>• Mining of this single-cell sequencing dataset for cytokine response pathways revealed that such macrophages may not only be primed in the lung to produce the cytokine triad, but may also respond to it	Vaccination did not lead to resolution of LC symptoms	**Viral reactivation**<br><br>**Immunological disturbance (including inflammation)**
**Yu 2022[82]**	51	0	28	28	0	23	23	- mMRC dyspnea score<br>- CAT score (used only the question related to dyspnoea)<br>- Subjective exertional	Pulmonary imaging using MRI scanning with dedicated imaging protocol and assessment of morphological imaging by	• Post-COVID group had a lower current self-reported physical activity<br>• Post-COVID group reported more dyspnea on	• No systematic structural changes identified on morphological lung MRI imaging	**Direct viral insult during the acute COVID event due to men having a higher expression of angiotensin-converting**

								impairment using self-reported Frändin-Grimby tool<br>- Objective exertional impairment using 6MWT	two radiologists independently. Also measured TTP (Time to Peak) and TTP ratio	CAT and mMRC, and shorter walking distance on 6MWT (both within normal range)<br>• Slower inflow of contrast bolus was noted to be significantly higher in Post COVID Group<br>• Mean TTP and TTP Ratio was worst in Post COVID males compared to Male controls<br>• For males, there was correlation between CAT scores with TTP and TTP ratios (6MWT did not correlate)<br>• Female post-COVID participants displayed greater variability in perfusion metrics relative to the female controls	• No correlation between time from symptom onset to MRI and any of the perfusion parameters<br>• Mean TTP and TTP Ratio did not differ in Post COVID females compared to female controls	**enzyme II-receptor, abundant in the lung, and a higher propensity for COVID-associated respiratory failure and mortality (hypothesised proof not provided)**
**Finlay 2022[88]**	11	Unknown	Unknown	7	Unknown	Unknown	4	Sense of smell (yes / no)	Gener expression RNA markers in nasal epithelial biopsies	• Olfactory epithelium contains T cells expressing inflammatory genes		**Immunological disturbance (including inflammation)**
**Talla 2022[63]**	493	Not stated	Not stated	204	Not stated	Not stated	289	Data merged into six major categories including:<br>- fatigue/malaise<br>- pulmonary<br>- cardiovascular<br>- gastrointestinal<br>- musculoskeletal<br>- neurologic<br>Other mild symptoms were combined into a single category as, "Any mild symptoms"	- Paired antibody proximity extension assays (PEA) and a next generation sequencing (NGS) readout to measure the relative expression of 1472 protein analytes per sample<br>- Analytes from the inflammation, oncology, cardiometabolic, and neurology panels were measured<br>- Flow cytometry was used to examine SARS-CoV-2-specific CD4+ and CD8+ T-cell responses using a validated ICS assay<br>- Gene Set Enrichment Analysis (GSEA) 24 was performed among genes that defined early acute infection status and genes that defined longitudinal changes	• IFN-y, IL-12 p40 and IFN-ydriven chemokines were consistently elevated within inflammatory LC clusters compared to non-inflammatory LC from clusters<br>• TNF, IL-6, and CCL7 remained persistently elevated	• LC patients distributed across inflammatory and non-inflammatory clusters<br>• Comparison of SARS-CoV-2 receptor binding domain (RBD)-specific IgG titers in infected subjects (LC + Recovered) 60 days PSO identified no significant difference between the inflammatory (4 and 5) and non-inflammatory clusters<br>• Comparison of SARS-CoV-2-specific CD4+ and CD8+ T cell frequencies between the inflammatory and non-inflammatory LC also did not show any significant difference	**Immunological disturbance (including inflammation)**
**Patterson 2021[71]**	Sample size appears to vary by variables assessed	Unclear	Unclear	26	Unclear	Unclear	20<br>(9 healthy / asymptomatic controls;	Grouped into:<br>- LC patients (symptoms at least 12 weeks and up to 15 months after infection)<br>- healthy controls	Monocyte levels by phenotype: - classical monocytes CD14++, CD16-<br>- intermediate monocytes CD14+, CD16+;	• Significantly higher level of intermediate monocyte for LC patients vs. healthy controls (but not vs. severe patients	• Only fragmented viral RNA was identified in a subset of five patients, though	**Viral persistence**<br><br>**Immunological disturbance (including inflammation)**

							11 severe COVID-19 cases)	- severe COVID-19 cases	- non-classical monocytes CD14lo, CD16+<br>And:<br>- Presence of SARS-CoV-2 RNA in peripheral blood mononuclear cells (PBMCs)	• Significantly higher level of non-classical monocyte for LC patients vs. healthy controls but not vs. severe patients<br>• SARS-CoV-2 S1 protein expressed in non-classical monocytes but not in classical or intermediate monocytes	multiple mutations were identified, ruling out persistent viral replication	**Endothelial dysfunction**
**Kravchenko 2021[81]**	83	0	41	41	0	0	42	Symptom burden questionnaire - no symptoms prescribed, so each participant filled in their own symptoms for acute C19 and LC. Each symptom rated 0-10. Symptoms listed:<br>Exertional dyspnea<br>Fatigue<br>Anosmia<br>Cardiac arrhythmia<br>Headache<br>Cough<br>Lymph node swelling<br>Chest pain<br>Fever<br>Muscle aches<br>Concentration problems	Multiparametric cardiac MRI	• In 3 (7%) LC participants, non-ischemic myocardial lesions were present, none in the control group (researchers concluded, however, that these are likely not the cause of the LC symptoms and could have been there due to unperceived myocarditis present prior to C19 infection);<br>• 6 (15%) LC participants had "incidental findings" (1 x small aberrant accessory lung, 1 x pericardial effusion, 1 x right ventricular high pressure overload, 1 x slight ventricular opacities in lower left lobe, 2 x discrete pleural effusions) that could have had an impact on prolonged cardiorespiratory symptoms, none found in control group (the researchers stated, however, that they cannot rule out that these were present prior to C19 infection).	LC participants did not demonstrate signs of active myocardial injury or inflammation	**None**
**Lee 2022[53]**	182 (48 with mild covid, 61 with severe covid, 73 covid negative controls)<br><br>(Breakdown of participants within the paper does not add up correctly.)	Unknown	Unknown	41	Unknown	Unknown	63	Self-reported health at three months including:<br>- dyspnea<br>- fatigue<br>- change in smell or taste	Epigenetics	• Only the comparison between the COVID-19 positive and COVID-19 negative individuals revealed differentially methylated CpGs at FDR<0.05 (specifically, cg22399236, cg03607951, and cg09829636). Findings support involvement of interferon responsive genes in the pathophysiology of COVID-19 and indicate a possible link to systemic autoimmune diseases.	• Analyses of COVID-19 severity and long-COVID did not identify any CpGs with significantly different methylation levels.<br><br>(No data presented from true control group.)	**Immunological disturbance (including inflammation)**
**Galan 2022[89]**	50	Unclear	Unclear	30	Unclear	Unclear	20	Demographic and clinical factors included in Random Forest algorithm (alongside immune response factors) to predict LC incidence:	Immune response factors as potential diagnostic biomarkers of LC included in Random Forest algorithm:	Statistically significant increase in:<br>• the level of regulatory T cells (Tregs) for LC group relative to recovered group	No significant difference in:<br>• total peripheral CD4+ T cells or in composition of	**Viral persistence**<br><br>**Immunological disturbance (including inflammation)**

								- gender<br>- O+ blood type<br>- lethargy<br>- pleuritic chest pain<br>- dermatological injuries<br>- mean body temperature<br>- dyspnea<br>- diarrhea<br>- conjunctivitis<br>- previous autoimmune diseases<br>- treatments during COVID-19 such as corticosteroids, antibiotics, vitamin D	- total NK cells (CD56+), CD3−CD56+CD16+, CD56+NKG2A−NKG2C+, and CD56+CD57+NKG2C+ subpopulations<br>- CD3+PD-1+<br>- total CD8+ T cells, CD8+<br>- TEMRA, and CD8±TCRgd+ subpopulations<br>- CD4+ Tregs<br>- cytotoxic activity against NK target cells K562 and/or SARS-CoV-2 infected Vero E6 cells	Significant increase in:<br>• level of CD8+ T cells for LC group relative to recovered group<br>• CD8+ TEMRA cells but not in other CD8+ T cell memory subpopulations<br>• CD8+TCRgd+ and CD8-TCRgd+<br>• total level of NK cells for LC group relative to recovered<br>• expression of CD16 marker on the surface<br>• NK cell subpopulation expressing the activation marker NKG2C for LC group relative to recovered<br>• population of activated memory NK cells<br>• cytotoxic activity of peripheral blood mononuclear cells (PBMCs) for LC group relative to recovered<br>• activation of caspase-3	CD4+ memory subpopulations between LC and recovered groups<br>• capacity of CD8+ T cells to release pro-inflammatory cytokines IFN-g, TNF-a and serine protease GZB<br>• expression of the immune exhaustion marker PD-1 in NK cells<br>• expression of the degranulation marker CD107a+<br>• NK cells not expressing CD16 marker or in NKT cell populations<br>• NK cell subpopulations with inhibitory markers NKG2A or KIR2DL5/CD158f<br>• total level of NK cells with memory marker CD57<br>Near significant difference in:<br>total level of CD3+ lymphocytes expressing the immune exhaustion marker PD-1 on the cell surface between groups	
**Sollini 2021 *(A)*[40]**	20	5	5	10	0	10	10	None reported	- FDG<br>- PET<br>- CT	Higher uptake in three areas: thoracic aorta, iliac arteries, femoral arteries	• No significant differences in total vascular score	**Endothelial dysfunction**
**Klein 2022[64]**	215 (4 groups).<br>99 in LC,<br>40 in HCM,<br>39 in CC,<br>37 in HCW group	Not clear	Not clear	99	Not clear	Not clear	39	- Fatigue<br>- Brain Fog<br>- Memory difficulty<br>- Confusion<br>- POTS	- Immune phenotyping<br>- SARS-CoV-2 specific humoral responses<br>- Glucocorticoids and soluble immune mediators<br>- Autoantibodies to the extracellular proteomes<br>- Humoral responses to distinct herpes viruses	• Serum cortisol was the most significant individual predictor of Long COVID status in the model- AUC of 0.96 (95% CI: 0.92-0.99)<br>In Long Covid participants:<br>• Immune phenotyping of circulating cell populations demonstrated specific elevations in both inflammatory and anti-viral immune responses (non-classical monocytes, B Lymphocytes)<br>• Significant decreases in circulating populations of cDC1 subsets, typically involved in cross-presentation to CD8+ T cells and Th1 response polarization	Autoantibody profiling indicated no stereotypical or shared extracellular autoantibodies that could differentiate participants with Long COVID from controls.<br><br>No correlation between the degree of autoantibody reactivity and Long COVID propensity, or any disproportionate targeting of functional pathways to distinguish Long COVID.<br><br>Prior hypotheses suggests autoantibodies may contribute to the	**Viral reactivation**<br><br>**Immunological disturbance (including inflammation)**<br><br>**Microclots**

										• Elevated SARS-CoV-2 specific humoral responses (S1 IgG levels; anti-N IgG)<br>- Altered humoral responses to distinct herpes viruses including the Epstein-Barr Virus minor viral capsid antigen gp23, the EBV fusion receptor component gp42, and the VZV glycoprotein E	pathogenesis of LC, results suggest they play a more limited role in disease pathology	
**Fancourt 2022[43]**	962	Not stated	Not stated	481	Not stated	Not stated	481	Depressive and anxiety symptoms using PHQ-9 and GAD-7. Covariates: Gender, ethnicity, age, education level, income, employment status.	PHQ-9, GAD-7 used to derive mental health growth trajectories, analysed using growth curve models.	• Long Covid as self defined was more common in those who reported depressive symptoms at infection (35% LC v 18% not LC)<br>• Anxiety became more common in the LC group with time	Anxiety did not influence the development of LC.	**Nothing concrete from this study - only hypothesising on proposed pathophysiology**
**Grist 2022[56]**	36	12	11	23	0	13	13	- Breathlessness questionaires<br>- 1 min. sit-to-stand test	- Hp-Xe MRI<br>- CT<br>- Lung function tests	• Hyperpolarized Xenon MRI and TLco demonstrate significantly impaired gas transfer in non-hospitalised Long Covid patients despite normal CT scans<br>• Abnormalities present many months after initial Covid infection<br>• Significant difference in TLco between non-hospitalised (78 ± 8%) and hospitalised patients (86 ± 9%)<br>• Significant differences in RBC:TP mean between healthy controls and hospitalised/non-hospitalised patients<br>• A lower figure of RBC:TP suggests infection with SARS-CoV-2 may have induced microstructure abnormality, causing a reduction in blood volume e.g. due to microclots and/or thickening of the alveolar membrane, both of which would be expected to cause a reduction in diffusing capacity	No significant differences in mean FEV or FVC between non-hospitalised and hospitalised patients	**Organ damage**
**Dennis 2022[44]**	628	70	466	536	0	0	92	- Breathlessness<br>- Cognitive dysfunction<br>- QoL across 5 health dimensions (mobility, self-care, usual activities, pain and discomfort, and anxiety and depression)	Link of symptoms to organ impairment (Heart, Lungs, Pancreas, kidney, liver, spleen)	• Multi-organ impairment at 6- and 12- months in 59% of individuals with Long COVID, with persistent symptoms and reduced function	• Despite some associations between organ impairment and symptoms, the study found insufficient evidence for distinct Long COVID subtypes<br>• Blood biomarkers, the current	**Organ damage (Overall weak link and no clear cause to effect relationship)**

											standard of care, showed no relation to clinical presentation<br>• Symptoms, blood investigations and quantitative, multi-organ MRI did not predict trajectory or recovery	
**Roca-Fernandez 2022[42]**	534	51	411	462	Unclear	Unclear	72	Standard range of WHO symptoms	- MRI scan of: Heart, Lung, Liver Pancreas, Spleen, Kidney<br>- Bloods: NTProBNP	• At 6 months 62/102 individuals with cardiac impairment had severe Long Covid based on questionnaires. Fatigue, SOB and chest pain most common<br>• In those with cardiac impairment, acute COVID-19 hospitalisation was associated with severe symptoms, wheezing, T1 elevation and multi-organ involvement, particularly kidney impairment), compared with non-hospitalised individuals<br>• At 12 months cardiac impairment persisted in 58%	• Cardiac involvement did not predict symptom severity<br>• No blood tests were abnormal	**Direct organ damage**
**Peluso 2022 *(B)*[90]**	280	43	165	208	6	66	72	Unclear	Viral titres	• A higher proportion of participants who experienced LC or LC>5, compared with those without LC, had EBV NA IgG levels greater than the limit of quantitation of 600 U/mL	• Only 1/50 participants had detectable plasma EBV DNA, and the level was below the limit of quantitation<br>• No association found with CMV	**Viral reactivation**
**Durstenfeld 2022 *(B)*[50]**	46	0	25	25	0	21	21	A composite symptom variable for cardiopulmonary PASC including chest pain, dyspnea, or palpitations.	- Echocardiography<br>- Troponin-I, CRP, NT-ProBNP, IL-6, IL-10, GFAP, MCP-1, NfL, IFN-gamma RBD-IgG,<br>- Cardiac MRI<br>- Ambulatory rhythm monitoring<br>- CPET	• Reduced exercise capacity on CPET due to chronotropic incompetence in LC group. Nineteen participants had AHRR<80%, which was associated with reduced exercise capacity and 4.9 ml/kg/min lower peak VO2<br>• On ambulatory rhythm monitoring, those with chronotropic incompetence during CPET had a higher average heart rate, higher minimum heart rate, lower maximum heart rate, and lower heart rate variability<br>• Markers of inflammation in the blood (hsCRP, IL-6, TNF-Œ±) and SARS-9 CoV-2 RBD IgG level measured at 3-9 months after infection are highly negatively	• Normal cardiac structure and function on CMRI, no arrhythmias	**Endothelial dysfunction**<br><br>**Autonomic dysfunction**<br><br>**Sinoatrial node dysfunction**

										correlated with peak VO2 more than one year after infection<br>• Among those with reduced compared to preserved exercise capacity, SARS-CoV-2 IgG and TNF-5α were significantly higher at the early time point (<90 days) with similar patterns in MCP-1		
**Maes 2022[55]**	125	unclear	unclear	86	unclear	unclear	39	Mental health structured interview	- IL-6<br>- Oxidative stress<br>- Insulin resistance<br>- Neurotoxicity	• Individuals with Long COVID had significantly lower total HR-QoL scores as well as physical, psychological, and environmental QoL scores (but not social QoL scores)<br>• Biomarkers of acute and Long COVID strongly predict lower WHO-QoL scores in Long COVID<br>• SARS-CoV-2 infection and the severity of the immune-inflammatory response during the acute phase on HR-QoL were significantly mediated by increased neurotoxicity and decreased calcium		**Direct Neuropsychiatric effect**
**Visvabharathy 2021[76]**	111	8	48	56	Unclear	unclear	55	Cognitive function	RNA-Seq analysis of CD4+ T cells	• Nucleocapsid-specific IgG titers were significantly elevated in Neuro-PASC compared with COVID convalescents<br>• Significant positive correlation between the magnitude of IFN-y production to N protein and higher pain interference scores and depression scores<br>• Neuro-LC patients have elevated IFN-y responses to internal proteins of SARS-CoV-2 (N and M proteins), enhanced activation of Tfh cells linked to increased anti-Nucleocapsid antibody production, but impaired CD8+ T cell memory compared with healthy COVID convalescents<br>• CD4+ T cells from Neuro-LC patients expressed lower levels of IL-6 and TNF- α in an antigen-specific manner relative to COVID convalescents following stimulation with nucleocapsid peptides		**Immunological disturbance (including inflammation); Neuropathology**

**Munker 2022[54]**	76	30	23	53	11	12	23	Symptoms: fatigue, cough, shortness of breath, dyspnoea on exercise, anosmia.	- Pulmonary function and cycle exercise blood gas analysis<br>- Anti SARS-CoV-2 IgG<br>- Chest CT<br>- Viral load (acute)	• Abnormal lung function (especially transfer factor) significantly lower in hospitalised patients<br>• Non-hospitalised patients showed hyperventilation	• Only 3.8% of patients had bronchial hyperactivity<br>• No relationship found between persistent symptoms and impaired lung function	**Interstitial lung disease**
**Peluso 2021 *(C)*[61]**	121	19	54	73	8	40	48	- 32 symptoms derived from the Centers for Disease Control and Prevention list of COVID-19 symptoms and from the Patient Health Questionnaire Somatic Symptom Scale<br>- Comparison between subgroups with and without LC at >90 day since infection	- IL-6; IFN-g; IL-10; IP-10; TNF-a; MCP-1; SARS-CoV-2 IgG	• Lack of significant difference in IL-6 between LC and non-LC groups at early-stage but significant difference at late-stage; significantly higher for LC patients<br>• Significantly higher IP-10 for LC patients than non-LC controls at early-stage, though not at late-stage; also, no longer significant when further adjusted for age, sex and hospitalisation<br>• Significantly higher TNF- α for LC patients than non-LC controls at early-stage, though not at late-stage<br>• Sensitivity analysis comparing top quartile of LC patients and non-LC controls: Significantly higher IL-6, IFN-g, IL-10 and TNF-a at early-stage for LC patients; IL-6 no longer significantly higher at late-stage for LC patients.<br>• Among those with LC, levels of binding antibodies correlated with TNF-a, IFN-y and MCP-1 at early-stage; at the late-stage, levels of binding antibodies correlated with IL-6, TNF-a (r = 0.28, P = 0.16), and MCP-1. In the group without LC, these antibodies correlated only with IFN-y at early-stage and IP-10 at late-stage	• Lack of significant difference between LC and non-LC groups at both early- and late-stages for IFN-g, IL-10, MCP-1, and SARS-CoV-2 IgG<br>• No statistically significant differences in trajectories of markers between LC and controls according to mixed effects model	**Immunological disturbance (including inflammation)**
**Sollini 2021 *(B)*[47]**	39	Not stated	Not stated	13	Not stated	Not stated	26	Principal symptoms of LC recorded in each case by clinical history	- PET/CT	• Vascular binary pattern and diffuse bone marrow FDG uptake in long bones was present in long COVID patients, but their prevalence did not differ compared to melanoma patients<br>• Significant hypo metabolism in parahippocampal gyrus linked to fatigue symptoms	• No correlation between long COVID patients' baseline characteristics, duration or type of symptoms, laboratory tests and PET/CT findings except in patients who required oxygen	**Endothelial dysfunction**

											during the acute phase of infection	
**Guo 2022[58]**	421	Not stated	Not stated	181	Not stated	Not stated	185 + 55 unknown infection status	-Memory<br>-Language<br>-Executive functioning	Object cognitive tests<br>-Word List Recognition memory test<br>- Pictorial associative memory test<br>-Category fluency test<br>- mental rotation figure<br>- Wisconsin card sorting test<br>-number counting test<br>- relational reasoning test	• COVID-19 (irrespective of ongoing symptoms) showed association with reduced performance on a factor created from memory task variables, but not other cognitive task factors.<br>• Detailed analysis of individual variables showed increased reaction time on performance of a verbal memory task.<br>• Memory emerged as a significant factor with those with severe ongoing symptoms performing significantly worse than those that had recovered<br>• Performance and reaction time was significantly impacted on verbal memory particularly those who had severe symptoms. Those who has mild symptoms were worse than those who recovered.	• The Category Fluency word-finding task and verbal memory task showed a similar patterns, with main effects falling below the threshold for significance once multiple comparisons were accounted for, but pairwise analysis revealing a strong negative impact of severe ongoing illness on the ability to produce category words.<br>• Little to no effect of the COVID-19 infection on 2D Mental Rotation (assessing visuospatial memory).	**There are "objective" cognitive differences between those that have and have not experienced COVID-19 . It was found that these are related to the severity of ongoing illness and that they may be most pronounced in tests of verbal memory**
**Chudzik 2022[83]**	103	Not stated	Not stated	45	Not stated	Not stated	32+26	- Fatigue<br>(Other symptoms gathered but not relevant for purposes of this study other than to confirm long covid)	- Vascular circulation/metabolic regulation via epidermal cell metabolism (NADH fluorescence) during cardio-pulmonary exercise testing	• Chronic fatigue associated with post-COVID syndrome is comparable with transient fatigue caused by high intensity exercise, in terms of vascular effects<br>• Significant similarities seen in physiology between post-exercise athletes and post-covid patients (although exercise induced change is reversible after 1-3 hours of rest)	• Non-significant differences in female vs male normoxia oscillatory index suggesting reasons for increased incidence of long covid in females	**Endothelial dysfunction**
**Gorecka 2022[46]**	30	0	20	20	0	10	10	Fatigue<br>Chest pain<br>Dyspnoea<br>Palpitations	Measured cardiac injury through the use of cardiovascular magnetic resonance (CMR) imaging<br>By assessing myocardial energetic status, function, perfusion and tissue characteristics.<br><br>Numeric elevation in NT-proBNP values<br><br>Inflammatory markers	None	• No significant differences in cardiac structure and function	**None**
**Clark 2021[80]**	100	5	45	50	0	50	50	Cardiovascular symptoms:<br>Mild symptoms: anosmia, ageusia, headache, mild	- Cardiovascular magnetic resonance (CMR)<br>- Some physiological data	• Cardiovascular LC identified in 19/50 vs 0/50	None	**Heart function directly affected by SARS-CoV-2 (?)**

								fatigue, milk upper resp tract illness, mild GI illness<br><br>Moderate symptoms: fever, chills, myaligia, lethargy, dyspnoea, chest tightness<br><br>Severe symptoms: Covid 19 related hospitalisation.		(mainly reduced right ventricular ejection fraction)<br>• Myocarditis diagnosed in 4/50 vs 0/50<br>• Takotsubo cardiomyopathy in 1/50 vs 0/50<br>• Significant differences seen in dyspnea on exertion and chest pain		
**Crunfli 2022[73]**	182	0	81	81	Not stated	Not stated	101	- Battery of cognition tests in 61 of the SARS-CoV-2 patient group	- MRI scans<br>- Identification of SARS-CoV-2 spike protein in brain cells	• MRI findings indicate that cortical thickness atrophy is associated with neuropsychiatric symptoms (anxiety/depression) and cognitive impairment (due to damage in the orbitofrontal cortex) in COVID-19 patients with mild or no respiratory symptoms. Cortical thickness may be related to loss of smell/taste.<br>• SARS-CoV-2-infected astrocytes exhibited marked metabolic changes resulting in a reduction of the metabolites used to fuel neurons and build neurotransmitters. Infected astrocytes also found to secrete unidentified factors that lead to neuronal death. These events could contribute to the neuropathological alterations, neuropsychiatric symptoms, and cognitive impairment observed in COVID-19 patients.<br>• Pathway for infection initiated via the NRP1 receptor. Suggests NRP1 blocker might reduce SARS-CoV-2 effects?<br>• Major alteration in cell activity due to SARS-CoV-2 was decreased pyruvate & lactate. Also reduced glutamate & GABA and increased neuronal death.	None	**Neuropathology**
